# Lysoquinone-TH1, a New Polyphenolic Tridecaketide Produced by Expressing the Lysolipin Minimal PKS II in *Streptomyces albus*

**DOI:** 10.3390/antibiotics7030053

**Published:** 2018-06-28

**Authors:** Torben Hofeditz, Claudia Eva-Maria Unsin, Jutta Wiese, Johannes F. Imhoff, Wolfgang Wohlleben, Stephanie Grond, Tilmann Weber

**Affiliations:** 1 Institut für Organische Chemie, Eberhard Karls Universität Tübingen, Auf der Morgenstelle 18, 72076 Tübingen, Germany; torben.hofeditz@gmx.de; 2 Interfakultäres Institut für Mikrobiologie und Infektionsmedizin, Eberhard Karls Universität Tübingen, Auf der Morgenstelle 28, 72076 Tübingen, Germany; claudia_unsin@yahoo.de (C.E.-M.U.); wolfgang.wohlleben@biotech.uni-tuebingen.de (W.W.); 3 GEOMAR, Helmholtz-Zentrum für Ozeanforschung Kiel, Düsternbrooker Weg 20, 24105 Kiel, Germany; jwiese@geomar.de (J.W.); jimhoff@geomar.de (J.F.I.); 4 German Center for Infection Research (DZIF), partner site Tübingen, Auf der Morgenstelle 28, 72076 Tübingen, Germany; 5 The Novo Nordisk Foundation Center for Biosustainability, Technical University of Denmark, Kemitorvet bygning 220, 2800 Kongens Lyngby, Denmark

**Keywords:** lysolipin, minimal PKS II, cyclases, benz[a]naphthacene quinone, tridecaketide, aromatic polyketide, pentacyclic angular polyphenol, extended polyketide chain

## Abstract

The structural repertoire of bioactive naphthacene quinones is expanded by engineering *Streptomyces albus* to express the lysolipin minimal polyketide synthase II (PKS II) genes from *Streptomyces tendae* Tü 4042 (*llpD-F*) with the corresponding cyclase genes *llpCI-CIII*. Fermentation of the recombinant strain revealed the two new polyaromatic tridecaketides lysoquinone-TH1 (**7**, identified) and TH2 (**8**, postulated structure) as engineered congeners of the dodecaketide lysolipin (**1**). The chemical structure of **7**, a benzo[a]naphthacene-8,13-dione, was elucidated by NMR and HR-MS and confirmed by feeding experiments with [1,2-^13^C_2_]-labeled acetate. Lysoquinone-TH1 (**7**) is a pentangular polyphenol and one example of such rare extended polyaromatic systems of the benz[a]napthacene quinone type produced by the expression of a minimal PKS II in combination with cyclases in an artificial system. While the natural product lysolipin (**1**) has antimicrobial activity in nM-range, lysoquinone-TH1 (**7**) showed only minor potency as inhibitor of Gram-positive microorganisms. The bioactivity profiling of lysoquinone-TH1 (**7**) revealed inhibitory activity towards phosphodiesterase 4 (PDE4), an important target for the treatment in human health like asthma or chronic obstructive pulmonary disease (COPD). These results underline the availability of pentangular polyphenolic structural skeletons from biosynthetic engineering in the search of new chemical entities in drug discovery.

## 1. Introduction

Polyketides are a large family of structurally-diverse natural products, mainly produced by bacteria, fungi, and plants. Their biosynthesis is catalyzed by distinct enzymes, termed type I polyketide synthases (PKS), type II PKS, type III PKS or variants thereof [1]. They exhibit broad ranges of pharmacological properties for use in clinical applications [2].

Many bacterial aromatic polyketides, such as the clinically used tetracycline, are biosynthesized by type II polyketide synthases (PKS II). Each PKS II contains a minimal set of enzymes (minPKS) that is required to synthesize a polyketide chain of defined length usually primed by acetyl-CoA and extended with malonyl-CoA to polycyclic products [3]. A minimal PKS II consists of two β-ketoacyl synthases (KS_α_ and KS_β_) and one acyl carrier protein (ACP). KS_α_ is responsible for loading malonyl-CoA extender onto the PKS II system and also for the iterative Claisen condensations to extend the polyketide chain. KS_β_, also referred to as chain length factor (CLF), is contributing to control the polyketide chain length [4]. Diverse cyclization patterns convert the polyketones to an enormous variety of structural polycyclic skeletons [5].

The final bioactive polycyclic PKS II natural products are aromatic compounds and often arise from additional enzymatic conversions, among them cyclases (CYC) [6], reductases (KR) [7], oxidases [8] and decarboxylating enzymes [2,9] or amidotransferases [10] for insertion of nitrogen or glycosyl transferases [11].

**Figure 1:**
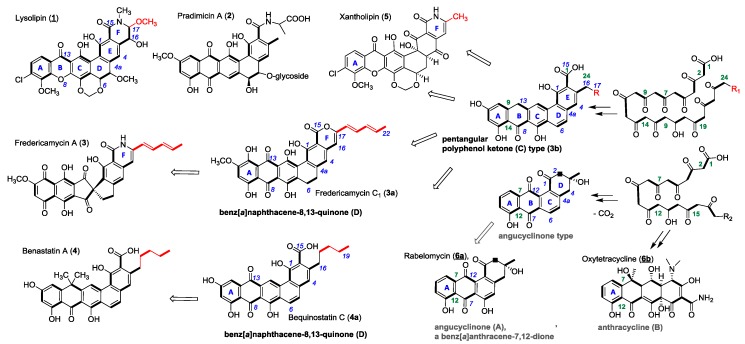
Chemical structures of PKS II products (**1**–**6**) and examples of biosynthetic congeners (**3a**, **b**, **4a**). PKS chains (acetate units bold) of angucyclinone (**A**) and anthracycline (**B**) type structures start to cyclize with ring A at C-7/C-12. Benz[a]naphthacene (**C**) and benz[a]naphthacene quinone (**D**) type structures cyclize starting with C-9/C-14 according to polyketide chain numbering (green). Variations of δ-substituents of ring-F highlighted in red. R = H or alkyl-, allylic carbon chains (usual chemical nomenclature in blue).

Lysolipin I (**1**) from *Streptomyces* Tü 4042 [12] and other members of microbial aromatic polyketides, such as pradimicin A (**2**) [13], fredericamycin A (**3**) [14], benastatin A (**4**) [15], bequinostatin C (**4a**) [16] and xantholipin (**5**) [17], are among the largest type II PKS products that have been described. They differentiate from the important groups of the smaller angucyclinones and anthracyclines (A-type, resp. B in Figure 1). They have distinct pentangular polycyclic aromatic core structures with pyridone, piperidone or lactone rings, respectively, added to a hexacyclic core structure as in xantholipin (**5**), lysolipin (**1**) or fredericamycins (**3**, **3a**) [18] (Figure 1). This additional ring F varies in δ-position with different substituents next to the amide. Several lysolipin derivatives have been described. While lysolipin I (**1**) carries a methoxy substituent at C-24, derivatives of lysolipin have been engineered which have a methyl group at this position (Patent WO2007079715 (A3), Combinature Biopharm, Berlin, Germany).

**Figure 2:**
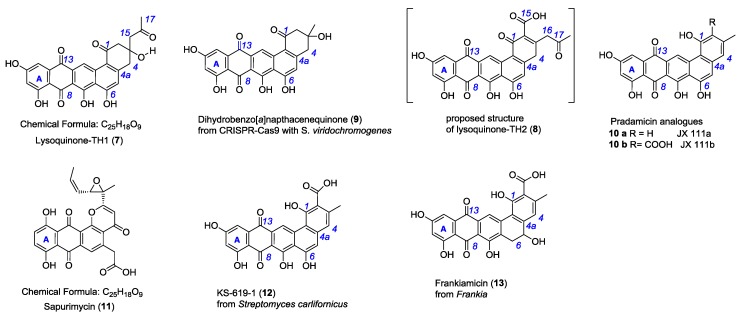
Chemical structure of lysoquinone-TH1 (**7**, identified), lysoquinone-TH2 (**8**, proposed), the constitutional isomer sapurimycin (**11**) and structurally related pentangular polyketides **9**–**13**.

Several biosynthetic gene clusters of these large PKS-II antibiotics are known and have been subject to extensive genetic and biochemical characterization [18,19,20,21,22,23]. In the biosynthetic pathways of pentangular polyaromatic polyketides, e.g., **1**–**5**, the respective cyclized polyphenols (**3b**) and polyphenolic quinones (**3a**) have been demonstrated as pathway intermediates; they have also been obtained as products of genetic engineering efforts (Figure 2). Therefore, we regard the class of pentangular quinones as the metabolic hub of the important aromatic polyketide products.

Here, we report that the heterologous expression of the lysolipin minimal PKS II genes, which comprise *llpF*, coding for the ketosynthase α (KSα), *llpE*, coding for the ketosynthase β (KSβ), and *llpD*, coding for the ACP, in combination with genes encoding the cyclases (*llpCI-CIII*) in the host *S. albus* J1074 resulted in the production of novel metabolites (Figure 2). The structure elucidation of the novel bioactive lysoquinone-TH1 (**7**), strong evidence for its tridecaketide backbone from the doubly labeled [1,2-^13^C_2_]-acetate-feeding experiments, and the potent bioactivity as potent phosphodiesterase inhibitor are discussed. 

## 2. Results and Discussion

### 2.1. Heterologous Production of Lysoquinone-TH 1 (**7**) in S. albus

The lysolipin gene cluster has been identified on a 42-kb genomic region in *Streptomyces tendae* Tü 4042 and analyzed by sequence comparison and heterologous expression in *S. albus* [21]. For this study, the genes coding for the minimal PKS II (*llpD-F*) and cyclases (*llpCI-CIII*) were amplified by PCR from the cosmid 4H04 encoding the complete lysolipin (**1**) gene cluster. The 4.1-kb PCR fragment was cloned into the vector pSET152ermE*p [24] under control of the constitutive promoter ermE*p yielding plasmid pCU1 (Figure S1). pCU1 was introduced into *S. albus* J1074 by intergeneric conjugation and the minimal PKS II and cyclase genes were heterologously expressed in culture. The recombinant strain expressing the minimal PKS and cyclases changed the color of the nutrient media (R5) and colonies on plates from yellowish to dark brown. In comparison, *S. albus* strain containing a pSET152ermE*p plasmid without insert does not show the phenotype.

Thus, this strain gained the ability to produce new substances. Thin layer chromatography (TLC) analysis revealed a strong red fraction proved to be not identical to lysolipin (**1**).

### 2.2. Isolation and Structure Elucidation of Lysoquinone-TH1 (**7**)

Starting from the newly observed colored product, we developed a work-up procedure towards the isolation of the pure compound for structure elucidation and biological profiling from a four liter fermentation broth in M65 medium. Acetone and methanol extractions removed water soluble and other unwanted compounds. Filtration with RP-18 material preceded the preparative HPLC purification using a C_4_ column and H_2_O (Ammonium formate, TFA): acetonitrile as solvents (see Supplemental Materials). Thereby, 3.4 mg of purified red compound (lysoquinone-TH1, **7**) were obtained from a four-liter fermentation broth from medium supplemented with sterile adsorber resin XAD-16 and subsequently characterized by TLC, LC-mass spectrometry (MS) and NMR-analysis (Figures S2–S5).

HR-ESI-MS data for the red metabolite **7** revealed a *m*/*z* = 461.087756 [M−H]^−^ ((Δppm = 0.11 ppm) and suggested the molecular formula for **7** to be C_25_H_18_O_9_ (M_R_ = 462,4). Consistently low resolution ESI-MS monitoring of the crude extracts exhibited *m*/*z* = 461.1 [M−H]^−^ and 463.2 [M+H]^+^) ions with stronger ionization in the negative mode. Thus, the sum formula for compound **7** proposed 17 degrees of unsaturation and implied a large aromatic ring system. 

The LC-MS/MS fragmentation analysis of lysoquinone-TH1 (**7**) gave evidence for a mass difference of *m*/*z* 58 with *m*/*z* = 403.1 [M−C_3_H_6_O-H]^−^ as the only fragmentation product, which was assigned to a McLafferty rearrangement and a neutral loss of acetone (Figure S2). These results indicated a stable substance in MS-fragmentation.

One- and two-dimensional NMR-data (^1^H-NMR, ^13^C-NMR, HMBC, HSQC, COSY) yielded ten proton signals; one aliphatic methyl-, one methylene group, four protons with methylene character and four aromatic protons (Figures S3–S5). The carbon to proton correlation (HSQC experiments) pointed to two isolated methylene groups with diasterotopic protons. These doublet protons (δ_H_ = 2.67, 2.91 and δ_H_ = 3.01, 3.25 ppm) underlined that they only couple within the methylene group, each (J = 15.9 Hz), and suggest a ring structure. Furthermore, a methyl group singlet (δ_H_ = 2.18 ppm) next to an aliphatic carbonyl group (δ_C_ = 207.8) implied no other direct substituents. However, this carbonyl group is adjacent to a methylene group (δ_H_ = 2.70, δ_C_ = 53.3 ppm), attached to a quaternary carbon (δ_C_ = 71.0 ppm) as part of the aliphatic ring system (C-1-C-4, C-4a, C-14b). The proton at δ_H_ = 9.48 indicated an aromatic proton with exceptional low field shift from two carbonyl groups (C-1, C-13) in spatial proximity. In consistence with the recorded ^13^C-NMR- and ^1^H-NMR-data, the HMBC-experiment unambiguously delivered assigned key correlations due to well separated signals which established the connectivity to the whole scaffold of lysoquinone-TH1 (**7**) and a full structural assignment (Table S1) to a pentangular polyphenolic core (Figure S4). Lysoquinone-TH1 (**7**) is a 3-(2-oxo-propyl)-decorated dihydrobenz[a]napthacene-8,13-quinone, a novel compound to the best of our knowledge.

Additionally, the purification protocol revealed another violet fraction with obviously a second compound (proposed as lysoquinone-TH2, **8**) not identical to lysolipin (**1**). However, only minute amounts were observed in production cultures, and presumably a distinct instability did not allow for purification and full structure elucidation. HR-ESI-MS data from extract fractions of the violet metabolite **8** revealed a *m*/*z* = 487.0670 [M−H]^−^ (Δppm = 0.1 ppm) and suggested the molecular formula for **8** to be C_26_H_16_O_10_ (M_R_ = 488.4). In comparison to **7**, 19 instead of 17 degrees of unsaturation were calculated. A large aromatic ring system is also concluded. The isolation of **8** was not achieved to allow for NMR studies. Thus, the analytical MS/MS and the UV-data of lysoquinone-TH1 (**7**) in addition to the knowledge of the mentioned McLafferty rearrangement are pointing to structure **8**. This proposed compound was named lysoquinone-TH2 (**8**) and resembles the C-2 carboxyl analogue of **7** as a yet unknown structure. 

In comparison to **7**, the only known constitutional isomer sapurimycin (**11**, C_25_H_18_O_9_) [25] is an annealed tetracyclic ring system. The rare moiety of a reduced ring E of angular naphthacene quinone of **7** is only known from metabolite **9**, generated via CRISPR-Cas9 technology with *S. viridochromogenes* [26]. JX111a (**10a**), JX111b (**10b**), and further precursors of pradimicin A (**2**) [27], KS-619-1 (**12**) [28], and frankiamicin (**13**) from *Frankia* [29] display a similar structural skeleton to lysoquinone-TH1 (**7**) and to the proposed structure lysoquinone-TH2 (**8**) (Figure 2). It could be anticipated that the benz[a]napthacene carbon skeleton originates from a native tridecaketide polyketide chain for the lysoquinones **7** and **8** from the min PKS while the parent natural product of strain *S.* Tü4042 lysolipin I (**1**) has a dodecaketide backbone.

### 2.3. Feeding Experiment with [1,2-^13^C_2_]-Labeled Acetate

**Figure 3:**
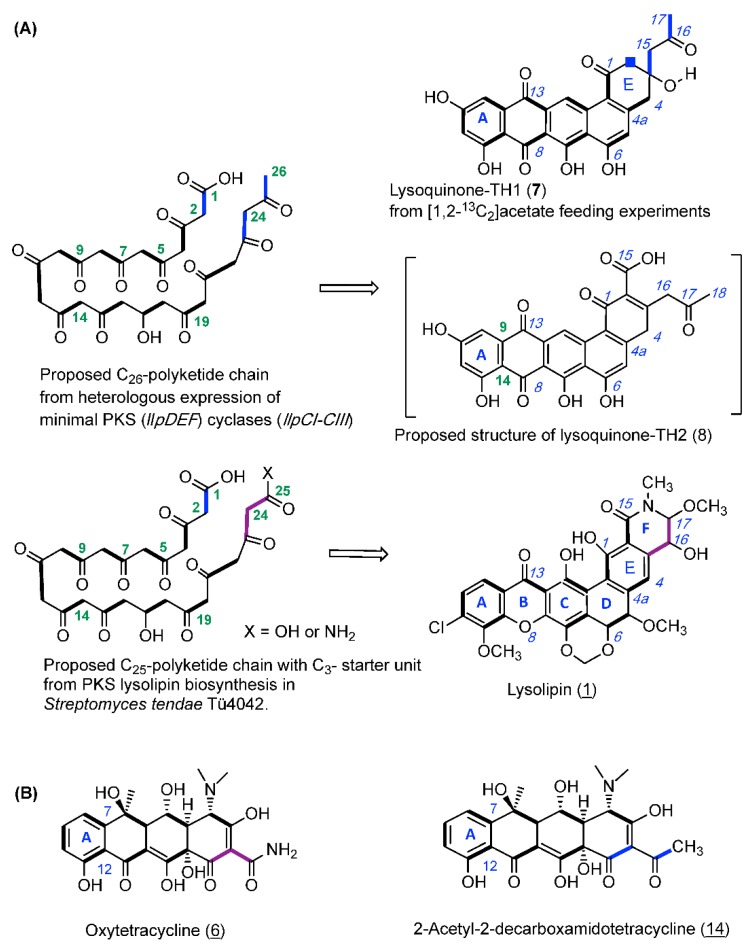
(**A**) Biosynthetic origin of lysoquinone-TH1 (**7**) and the proposed structure of lysoquinone-TH2 (**8**): Biosynthesis hypothesis from feeding experiments with doubly labeled [1,2-^13^C_2_] acetate with the *S. albus* host strain and heterologous expression of the lysolipin minimal PKS genes (*llpD-F*) and cyclase genes (*llpCI-CIII*). (**B**) Oxytetracycline (**6**) from a biosynthesis primed with malonamide in the wild-type producer, and with two acetate units in the mutant producer.

For validating the biogenesis of lysoquinone-TH1 (**7**) a feeding experiment with the doubly ^13^C-labeled [1,2-^13^C_2_] acetate was carried out, and lysoquinone-TH1 (**7**) was purified from the culture and subjected to NMR spectroscopy. All carbon atoms turned out to be enriched [30] with highly specific incorporation rates between 2.0 and 7.3 (Figure 3, Figure S4 and S6, Table S1). The specific coupling constants from NMR analysis again confirmed the structure of lysoquinone-TH1 (**7**) and suggest a polyketide origin assembled from 13 acetate extender units (tridecaketide, Figure 2). It is therefore among the largest polyketides formed by a minimal PKS II (LlpD, E, F) with cyclases (LlpCI–CIII) via heterologous expression. It could be anticipated that the proposed structure of lysoquinone-TH2 (**8**) further corroborates the native tridecaketide precursor and carries the additional carboxyl group (C-15) of the final extender acetate unit. The continuous labeled-acetate chain of **7** corresponds with the pattern of the further post-PKS-processed antibiotic lysolipin I (**1**) [31]. 

A specific feature of the lysolipin polyketide biosynthesis is the priming by a malonate-derived starter unit [31]. However, in the identified lysoquinone-TH1 (**7**) as well as in the proposed structure of lysoquinone-TH2 (**8**), two acetate units replace the original C_3_-malonyl/malonamide starter unit. Respective observations were also made for oxytetracycline (**6b**) with a malonamide starter unit since heterologous expression of the oxytetracycline minimal PKS of *Streptomyces rimosus* in *S. coelicolor* yielded tetracycline analogue **14** with two acetate units priming the polyketide backbone instead [32] (Figure 3). Co-expressing the *oxyD* gene with the oxytetracyline minimal PKS in heterologous expression experiments has shown that it is presumably responsible for generating the characteristic malonamate starter unit. OxyD codes for an amidotransferase and is homologous to the putative amidotransferase LlpA of lysolipin biosynthesis. Therefore, priming of the PKS biosynthesis with malonamate and and formation of a N-heterocyclic product does not require additional enzymes [10].

Studies on hybrid PKS pathways with benastatin A (**4**) revealed isolated hybrid PKS-II products with the number of extender units increased if shorter starter units were used. In analogy, based on the observations on the identified lysoquinone-TH 1 (**7**) and the proposed structure of lysoquinone-TH2 (**8**) it can be anticipated that the number of elongation steps is dependent on the length of the polyketide chain [33] fitting into the substrate pocket of the KSα/KSβ-complex.

### 2.4. Biological Activity of Lysoquinone-TH1 (**7**)

Lysolipin (**1**) is highly active against various Gram-positive bacteria and shows antifungal activity. While the molecular target of lysolipin (**1**) still remains elusive, there is strong indication that this xanthone antibiotic targets the bacterial cell envelope [12,34].

Because of the high antibiotic activity of lysolipin (**1**) in nM-range, biological assays with lysoquinone-TH1 (**7**) were performed. Only weak antibiotic activities at 100 µM concentration of lysoquinone-TH1 (**7**) were observed against the Gram-positive strains *Staphylococcus lentus*, *Staphylococcus epidermidis* and *Propionibacterium acnes* with inhibition of 39%, 66% and 74%, respectively, in comparison to the positive control chloramphenicol [35]. Therefore, no IC_50_ values were determined.

KS-619-1 (**12**) and K-259-2 are representing inhibitors of the cyclic nucleotide phosphodiesterase (PDE4) [28,36,37,38]. The enzyme PDE4 is a very attractive target for the treatment of asthma, chronic obstructive pulmonary disease (COPD), psoriasis, schizophrenia, diet induced obesity, glucose intolerance and multiple sclerosis [39,40,41,42,43,44]. PDE4 addresses cyclic nucleotides like cAMP and cGMP and degrades these cellular messengers. However, these messengers possess regulatory functions in almost all cells so it is regarded an important target [45], therefore, detailed studies with different inhibitors are needed to evaluate an ideally selective bioactivity.

For profiling lysoquinone-TH1 (**7**), an enzyme assay using PDE-4B2 was carried out according to Schulz et al. [39]. A clearly defined IC_50_ value could not be determined in this assay due to an additional luminescence signal derived from the chromophore of lysoquinone-TH1 (**7**), an extensive polyaromatic skeleton. However, an IC_50_ range of 10–20 µM could be inferred from the assays against the PDE-4B2 enzyme. The MIC (minimal inhibition concentration) of lysoquinone-TH1 (**7**) was determined to a value of 2.33 µM (±0.04) corresponding to 3.368 log(nM) (±0.007) (Figure S7). The standard used in these assays was rolipram (IC_50_ = 0.8 µM (±0.1)) [39], an optimized and approved drug. Lysoquinone-TH1 (**7**), a completely new substance, is only 10-fold less active as this well-known PDE4 inhibitor rolipram. When lysolipin (**1**) was tested in the same assay, no PDE4 inhibition was detected up to a concentration of 50 µm.

## 3. Materials and Methods

### 3.1. Cloning of the Lysolipin Minimal PKS

The genes of the lysolipin minimal PKS II (*llpD*, *llpE*, *llpF*), which are surrounded by the cyclases *llpCI*, *llpCII*, and *llpCIII,* were amplified by PCR using the primers minPKScyc-fw-Hind (aaa gct tga gta gcc aaa cgg gtt c) and minPKScyc-revSs (aag aat tca ata ttg tgc cca cca gta cac) and the template cosmid 4H04 [21] using ProofStart PCR polymerase kit (*Qiagen*). The PCR Program used in an PTC-100 thermocycler (MJ Research, Waltham, MA, USA) was: 95 °C—5 min; 30 cycles with (94 °C—90 s, 62 °C—90 s, 72 °C—4 min); 72 °C—10 min. The PCR product was then cloned into pSETermE*p [24] (Combinature Biopharm AG, Berlin, Germany) via the EcoRI/HindIII restriction sites. The resulting plasmid pCU1 was checked by restriction and DNA sequencing. 

The plasmid pCU1 was introduced into *Streptomyces albus* J1074 via a standard intergeneric conjugation protocol as, for example, described in [46].

### 3.2. Culture Conditions

A pre-culture with medium G20 (600 mL) in six 300 mL Erlenmeyer flasks was inoculated with *S. albus* J1074 and apramycin (50 µg/mL) for 48 h at 28 °C and 180 rpm (B. Braun Certomat HK with shaker B. Braun Certomat U, B. Braun, Melsungen, Germany). The main culture was inoculated with 400 mL of the pre-culture and was grown up in medium M65 (3.6 L) under selection with apramycin (50 µg/mL) in a fermenter (B. Braun Biostat B, B. Braun, Melsungen, Germany) for 96 h at 28 °C and 300 rpm. After 24 h, 15 g/300 mL of sterile XAD-16 was added to the culture. Nutrient solutions: G20 (Glycerol (20 g), malt extract (10 g), yeast extract (4 g) in 1 L of tab water. pH = 7.2). M65 (Malt extract (10 g), yeast extract (4 g), D-glucose (4 g), CaCO_3_ (2 g) in 1 L of tab water. pH = 7.2).

### 3.3. Extraction and Isolation

For initial detection, agar plates from *S. albus* incubation were extracted with ethyl acetate, the organic phases evaporated and the extract applied to silica gel TLC analysis (solvent cyclohexane/ethylacetate/methanol 6:8:1, with 1% of trifluoric acetate acid added). For purification, 4 L of culture broth with XAD-16 was filtered over Celite to separate the mycelia from the liquid culture. The filtrate was autoclaved and discarded. The mycelia were extracted two times with acetone/methanol 7:3 and then in acetone/methanol 1:1 in an ultrasonic bath. After filtration, the organic phases were combined, evaporated, water was added, and the pH adjusted to 4–5 with 1 M HCl. Extraction with ethyl acetate and evaporation gave the crude extract. RP silica gel was pretreated with 3–4 column volumes (CV) of pyridine and washed with 3–4 CV of water as a basic activation of the RP phase. After column conditioning with 1–2 CV of the solvent acetone/methanol 1:1 the extract was loaded, and red and violet fractions were selected, accompanied by TLC analysis on silica gel (see above) and LC-ESI-MS analysis (HPLC, Agilent 1100 series. Ion trap, Bruker Daltonic Esquire 3000+, He as reactant gas, with Data Analysis software, Bruker Daltonik, Bremen, Germany). Combined red fractions were dissolved in DMSO and purified with HPLC (Thermo Ultimate 3000 Thermo Scientific, Dreieich, Germany); Column: Dr. Maisch, Ammerbuch-Entringen, Germany, Reprosil 120 C-4, 5 µm, 250 × 20 mm id, pre-colum: Dr. Maisch, standard guard Reprosil 120 C-4, 30 × 20 mm id, flow rate: 13.0 mL/min.; program: 20 min at 45% B, in 5 min to 75% B, 8 min at 75% B, in 2 min to 45% B, 8 min at 45% B; solvent: A = ammonium formate (20 mM) + 0.1% TFA in water; B = acetonitrile). A retention time from 12 to 13 min was observed. After evaporating a Sephadex LH-20 (2 × 2 cm, methanol) and following extraction three times with ethyl acetate and three times with diisopropyl ether was necessary for desalting the sample. This work up procedure gives 3.4 mg lysoquinone-TH1 (**7**) from four liters of culture.

### 3.4. Feeding Experiment with [1,2-^13^C_2_]-Labeled Acetate

For the feeding experiment 2.0 g of the doubly labeled [1,2-^13^C_2_] acetate (99% enrichment; Cambridge Isotope Laboratories, Inc., Tewksbury, MA, USA), which corresponds to 5.95 mm end concentration in the fermenter (4 L, B. Braun Biostat B) were added to the culture broth after 32 h of cultivation. The isotope labeled lysoquinone-TH1 (**7**) was purified with same protocol as described above and subjected to NMR-analysis (^13^C-NMR, ^1^H-NMR, HMBC, HSQC).

### 3.5. Biological Activity Assays

Antibacterial assays were carried out with the test strains *Staphylococcus lentus* DSM 6672, *Staphylococcus epidermidis* DSM 20044 and *Propionibacterium acnes* DSM 1897 using a cell viability test based on the reduction of resazurin to resorufin. Details on the cultivation conditions of the strains *S. epidermidis* and *P. acnes*, as well as on the evaluation of cell viability are described by Silber et al. [34]. The experiments with *S. lentus* were performed in the same manner as *S. epidermidis*. The positive control chloramphenicol was applied in a concentration of 10 µM for *S. lentus* and *S. epidermidis* and of 1 µM for *P. acnes*.

The effect of lysoquinone-TH1 (**7**) on PDE-4B2, a human recombinant cyclic adenosine monophosphate (cAMP) specific phosphodiesterase (BPS Bioscience no. 60042, San Diego, CA, USA) was determined in 96 well plates using the PDELight HTS cAMP Phosphodiesterase Kit (Lonza, LT07-600, Wuppertal, Germany). Lysoquinone-TH1 (**7**) was diluted in 50 mM Tris-HCl buffer (pH 7.5) containing 8.3 mM MgCl_2_ and 1.7 mM EGTA. 10 µL of each dilution was transferred to a well. 20 µL PDE-4B2 solution (0.25 U/µL) were added. The reaction was started by adding 10 µL of 12 mM cAMP (Sigma A9501, Taufkirchen, Germany) dissolved in 50 mM Tris-HCl buffer (pH 7.5) containing 8.3 mM MgCl_2_ and 1.7 mM EGTA to each well of the microtiter plate. PDE-4B2 hydrolysed cAMP to adenosine monophosphate (AMP). After an incubation at 30 °C for 30 min, the reaction was stopped by transferring 30 µL solution containing 10 µL PDELight Stop Solution and 20 µL PDELight AMP detection reagent. The detection reagent converted AMP to ATP and luciferase catalyzed the formation of light from ATP and luciferin. The emitted light is proportional to the level of AMP produced. AMP was quantified after incubation at 30 °C for 10 min by measuring the luminescence using the microtiter plate reader Infinite M200 (Tecan, Crailsheim, Germany) with 0.1 s integration time. The assays were performed in duplicates. Rolipram (4-[3-(cyclopentyloxy)-4-methoxyphenyl]-2-pyrrolidinone) was used as positive control.

## 4. Conclusions

Lysoquinone-TH1 (**7**) is a “non-natural natural product”, a pentangular aromatic polyketide derived from engineering of the lysolipin biosynthetic pathway. It was produced with *Streptomyces albus* as host expressing the minimal PKS II genes (*llpD-F*) in combination with three cyclases (*llpCI-CIII*) of the lysolipin gene cluster. In a bioactivity profiling study, it was shown that lysoquinone-TH1 (**7**) only has weak antibacterial activity, but instead is an inhibitor of phosphodiesterase 4 (PDE4) which is a target for treatment of pulmonary diseases. The lysoquinone-TH1 (**7**) biosynthetic pathway, which was deduced based on NMR data and supported by the new but postulated analogue lysoquinone-TH2 (**8**) also provides insights on the biosynthesis of lysolipin I (**1**). Evidence is given for the acetate-derived tridecaketide backbone of **7** in contrast to the dodecaketide malonyl-derived (malonyl- or malonamide CoA) precursor chain of the parent compound lysolipin I (**1**).

## Supplementary Materials

The following are available online at http://www.mdpi.com/2079-6382/7/3/53/s1. Figure S1: Plasmid map of pCU1; Figure S2: HPLC and LC-MS of lysoquinone-TH1 (7) and proposed lysoquinone-TH2 (8); Figure S3: NMR spectra of lysoquinone-TH1 (7); Figure S4: NMR spectra of 13C-enriched lysoquinone-TH1 (7); Figure S5: 2D-NMR data of lysoquinone-TH1 (7); Figure S6: ^13^C enrichment of 7; Figure S7: PDE-4B2 inhibition assay with lysoquinone-TH1; Table S1: Level of enrichment and specific incorporation of lysoquinone-TH1 (7), resulted from the feeding experiment with doubly labeled [1,2-^13^C_2_] acetate.
