# Supplementary material for: Lysoquinone-TH1, a New Polyphenolic Tridecaketide Produced by Expressing the Lysolipin Minimal PKS II in Streptomyces albus
Source: Antibiotics (Basel). 2018 Jun 28;7(3):53. doi: 10.3390/antibiotics7030053 (PMC6164072; doi:10.3390/antibiotics7030053)
Supplement: Supplementary file 1 [file antibiotics-07-00053-s001.pdf]

Supplemental Information

Lysoquinone-TH1, a new polyphenolic tridecaketide produced by expressing the lysolipin minimal PKS II in *Streptomyces albus*

Torben Hofeditz,[1] Claudia Eva-Maria Unsin[2], Jutta Wiese,[3] Johannes F. Imhoff,[3] Wolfgang Wohlleben[2,4], Stephanie Grond,[1*] and Tilmann Weber[2,5]*

[1]Eberhard Karls Universität Tübingen, Institut für Organische Chemie, Auf der Morgenstelle 18, 72076 Tübingen, Germany.
[2]Eberhard Karls Universität Tübingen, Interfakultäres Institut für Mikrobiologie und Infektionsmedizin, Auf der Morgenstelle 28, 72076 Tübingen, Germany.
[3]GEOMAR, Helmholtz-Zentrum für Ozeanforschung Kiel, Düsternbrooker Weg 20, 24105 Kiel, Germany.
[4]German Center for Infection Research (DZIF), partner site Tübingen, Auf der Morgenstelle 28, 72076 Tübingen, Germany
[5]The Novo Nordisk Foundation Center for Biosustainability, Technical University of Denmark, Kemitorvet bygning 220, 2800 Kgs. Lyngby, Denmark
*corresponding authors: Tilmann Weber: tiwe@biosustain.dtu.dk; Stephanie Grond: stephanie.grond@uni-tuebingen.de

Table of Contents

1 Figure S1: Plasmid map of pCU1 ... 2

2 Numbering of pentangular PKS II products ... 3

3 Physicochemical properties with NMR data of lysoquinone-TH1 (7) ... 4

4 Figure S2: HPLC and LC-MS of lysoquinone-TH1 (7) and proposed lysoquinone-TH2 (8) ... 5

5 Figure S3: NMR spectra of lysoquinone-TH1 (7) ... 7

6 Figure S4: NMR spectra of ^{13}C-enriched lysoquinone-TH1 (7) ... 9

7 Figure S5: 2D-NMR data of lysoquinone-TH1 (7) ... 13

8 Figure S6: ^{13}C enrichment of 7: Structure, Table S1 ... 16

9 Figure S7 – PDE-4B2 inhibition assay with lysoquinone-TH1 ... 18

1 Figure S1: Plasmid map of pCU1

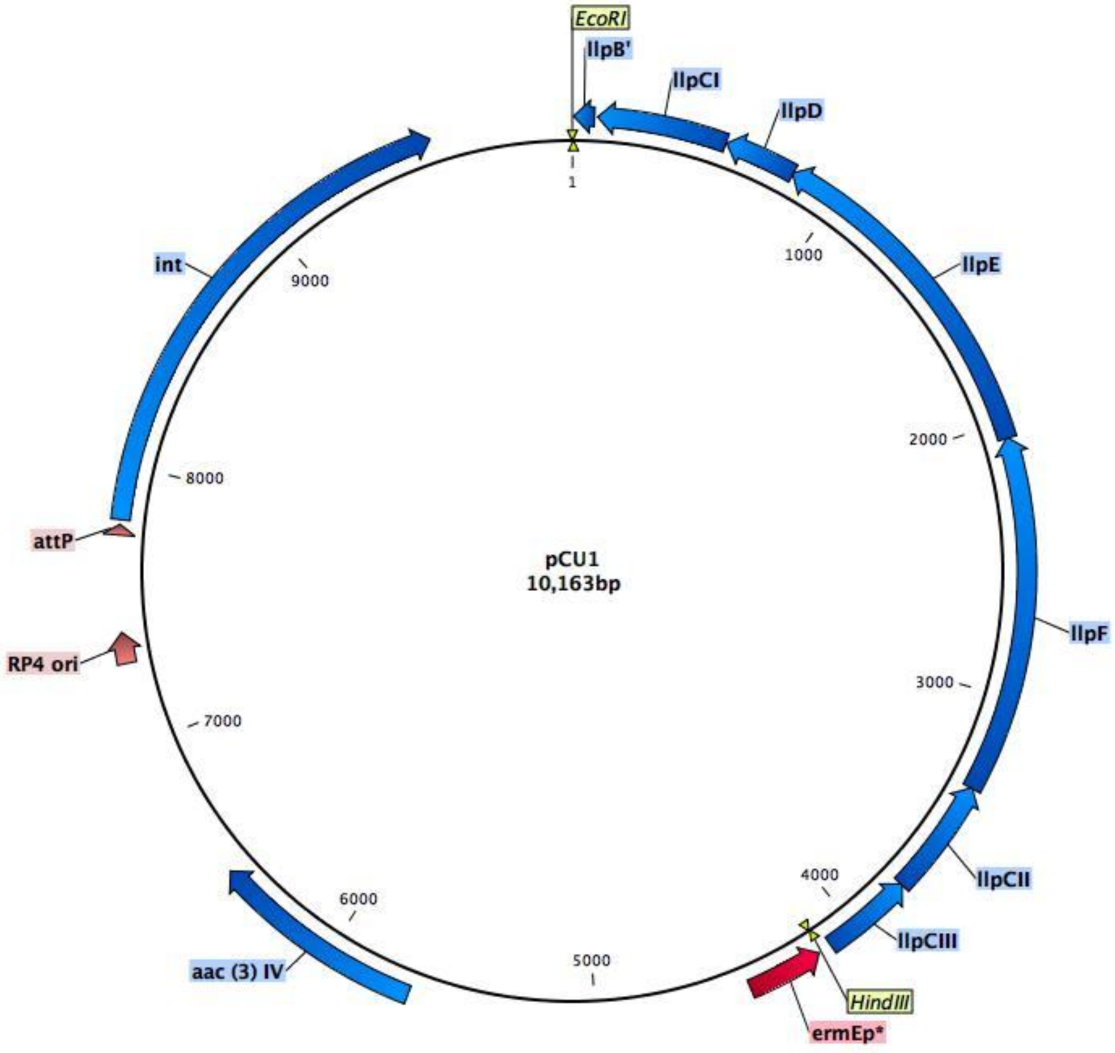

Figure S1: Plasmid map of pCU1. *aac(3)IV*: apramycin resistance gene; *int*: PhiC31 integrase gene; *llpCI-CIII*: polyketide cyclase genes; *llpF*: ketosynthase α gene; *llpE*: ketosynthase β gene; *llpD*: ACP gene; *llpB'*: Fragment of *llpB* gene coding for an hypothetical protein with unknown function; *ermEp**: *ermE** promoter; RP4ori: RP4 origin of transfer; *attP*: PhiC31 attachment site.

2 Numbering of pentangular PKS II products

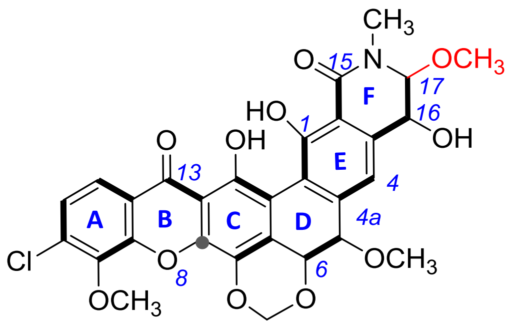

Lysolipin (<u>**1**</u>)
with classical chemical numbering (blue)

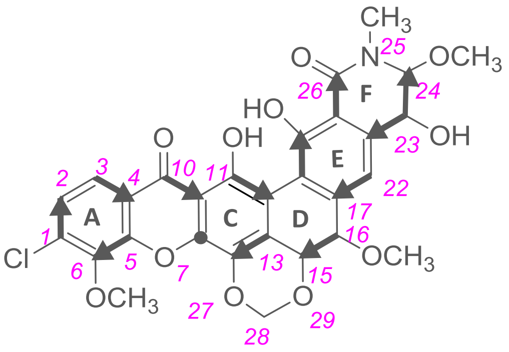

Lysolipin (<u>**1**</u>)
with former numbering,
originally from X-Ray analysis (see ref. 17, 21);
with polyketide chain deduced
from feeding experiments with ^{13}C-labeled acids
(●→ acetate, ⟵⟶ malonate, see ref. 31)

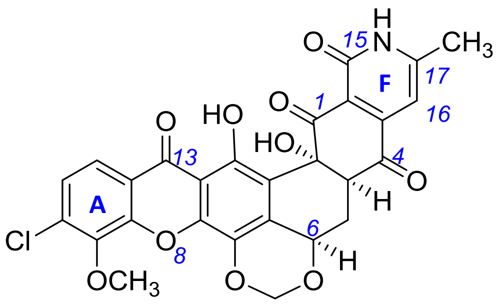

Xantholipin (**5**)
with classical chemical numbering (blue)

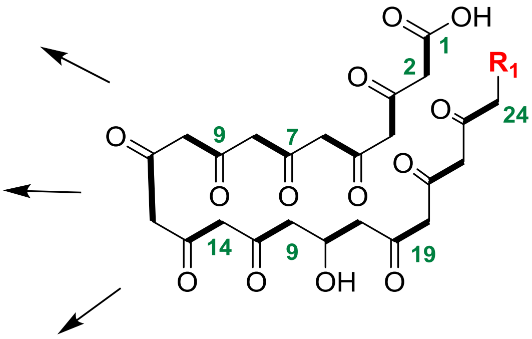

Dodecaketide-PKS-precursor
(PKS-biosynthetic numbering (**green**))

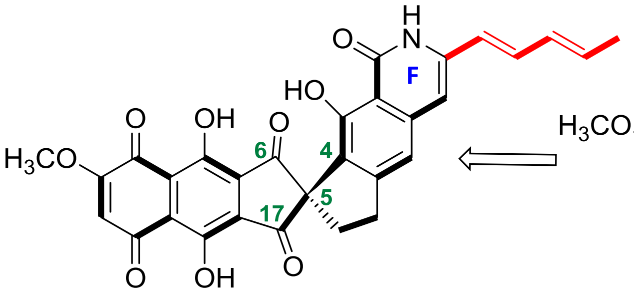

Fredericamycin A (**3**)
numbering due to PKS-biosynthesis
(see also ref. 18, Fig 2)

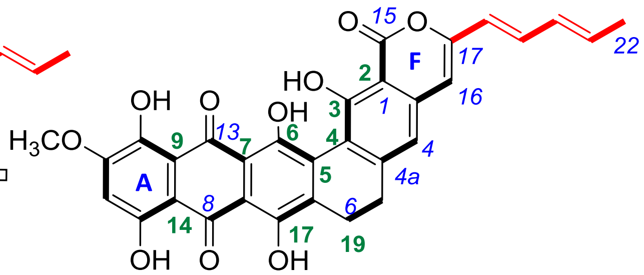

Fredericamycin C$_1$ (**3a**)
benz[*a*]naphthacene-8,13-quinone

3 Physicochemical properties with NMR data of lysoquinone-TH1 (7)

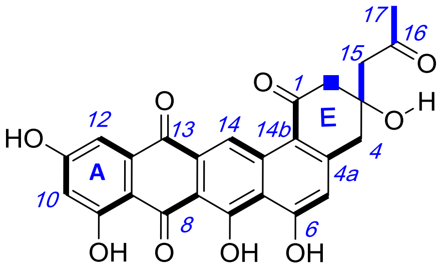

Lysoquinone-TH1 (**7**)
(from the doubly labeled
[1,2-$^{13}C_2$]acetate feeding experiments)

Lysoquinone-TH1 (**7**) was isolated as a red solid with following physical characteristics:

M_r = 462.10 g·mol^{-1} ($C_{25}H_{18}O_9$); **ESI-MS** (negative mode): *m/z* = 461.1 [M-H]$^-$. (positive mode): *m/z* = 463.2 [M+H]$^+$; **HR-ESI-MS** (calculated): *m/z* = 461.087806 [M-H]$^-$. (measured): *m/z* = 461.087756 [M-H]$^-$. (relative mass deviation = 0.11 ppm); **IR** (KBr): ῡ = 3418, 2955, 2921, 2851, 1731, 1714, 1617, 1455, 1375, 1358, 1272, 1172, 1117, 1073, 799 cm^{-1}; **UV (MeOH):** λ_{max} (ε) = 515 (1940), 397 (1512), 276 (6231), 204 (22475) nm. **(MeOH/HCl):** λ_{max} (ε) = 477 (2532), 284, (5853), 204 (22261) nm. **(MeOH/NaOH):** λ_{max} (ε) = 592 (789), 525 (1628), 395 (1447), 329 (3979), 282 (4127), 248 (6560), 210 (11986), 207 (12479), 203 (4423) nm. **^{1}H-NMR** (600 MHz, DMSO-*d*$_6$): δ = 2.18 (s, 3H, 17-H_3), 2.67 (d, *J* = 15.9 Hz, 1H, 2-H_a), 2.70 (s, 2H, 15-H), 2.91 (d, *J* = 15.9 Hz, 1H, 2-H_b), 3.01 (d, *J* = 15.9 Hz, 1H, 4-H_a), 3.25 (d, *J* = 15.9 Hz, 1H, 4-H_b), 6.55 (d, ^3J = 2.6 Hz, 1H, 10-H), 6.66 (s, 1H, 5-H), 7.09 (d, 1H, ^3J = 2.6 Hz, 12-H), 9.48 (s, 1H, 14-H) ppm. **^{13}C-NMR** (150.6 MHz, DMSO-*d*$_6$): δ = 32.4 (C-17), 42.8 (C-4), 52.2 (C-2), 53.3 (C-15), 71.0 (C-3), 107.3 (C-6a), 108.0 (C-12), 108.7 (C-10), 110.3 (C-8a), 114.4 (C-5), 116.2 (C-7a), 117.7 (C-14b), 118.3 (C-14), 131.4 (C-13a), 135.6 (C-12a), 136.9 (C-14a), 150.6 (C-4a), 164.3 (C-7, C-9), 164.5 (C-6, C-11), 182.2 (C-13), 186.6 (C-8), 196.2 (C-1), 207.8 (C-16) ppm.

No CD-effect or angle of rotation was assessible.

4 Figure S2: HPLC and LC-MS of lysoquinone-TH1 (7) and proposed lysoquinone-TH2 (8)

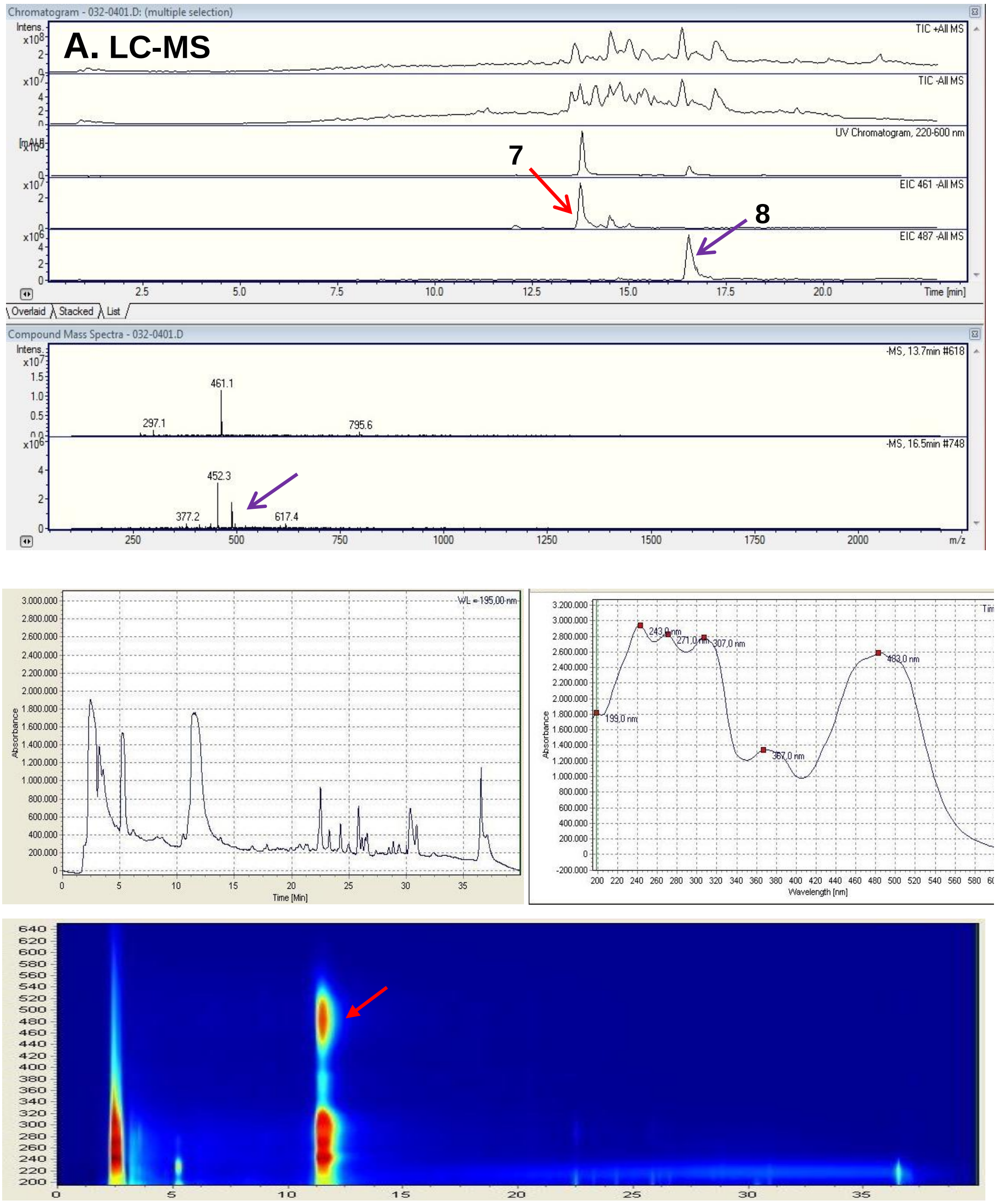

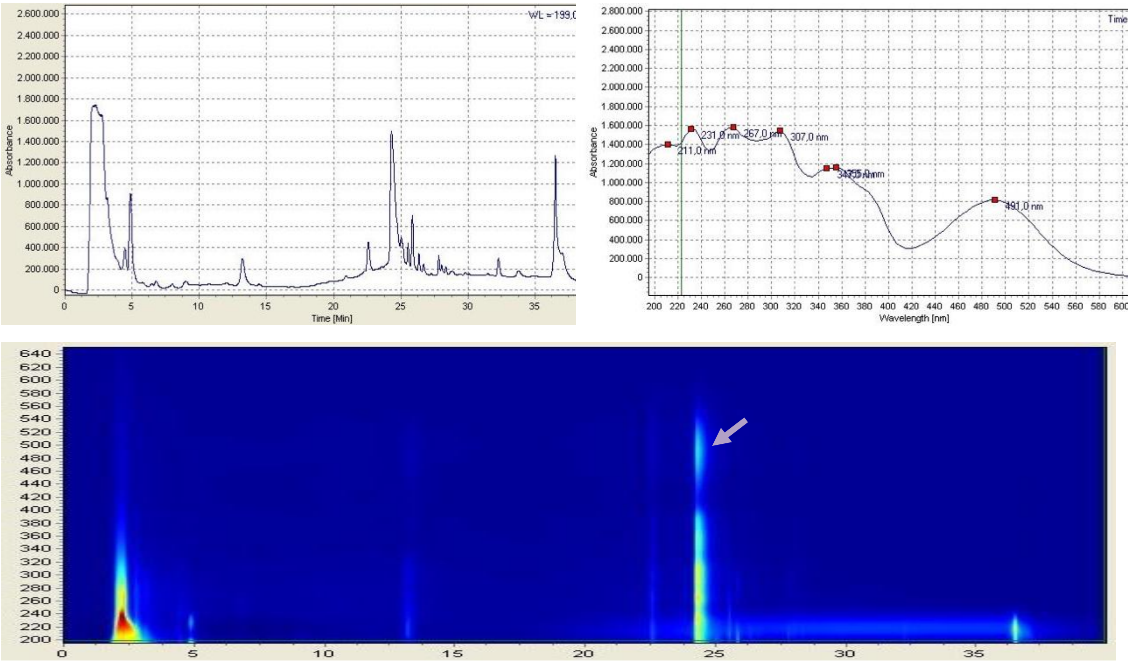

<u>Figure S2:</u> **A:** LC-MS chromatogram of a pre-purified fraction of colored pigments. **7** R_t = 13.7 minutes; *m/z* = 461.1 $[M-H]^-$ (red arrow). **8** R_t = 16.5 minutes; *m/z* = 487.1 $[M-H]^-$ (violet arrow). **B:** HPLC-UV-DAD (diode array detector) chromatogram of the extract with lysoquinone-TH1 (**7**, red arrow) measured with an acid additive (at wavelength of 200 nm). **C:** Extracted UV spectrum of red pigment **7**. **D:** UV-DAD chromatogram of wavelengths of 190–650 nm shows lysoquinone-TH1 (**7**, red arrow). **E:** HPLC-UV-DAD chromatogram of the extract with lysoquinone-TH2 (**8**, violet arrow), measured with an acid additive (at a wavelength of 200 nm). **F:** Extracted UV spectrum of violet pigment **8**. **G:** UV-DAD chromatogram of wavelengths of 190–650 nm shows the violet pigment **8** (violet arrow).

5 Figure S3: NMR spectra of lysoquinone-TH1 (7)

A

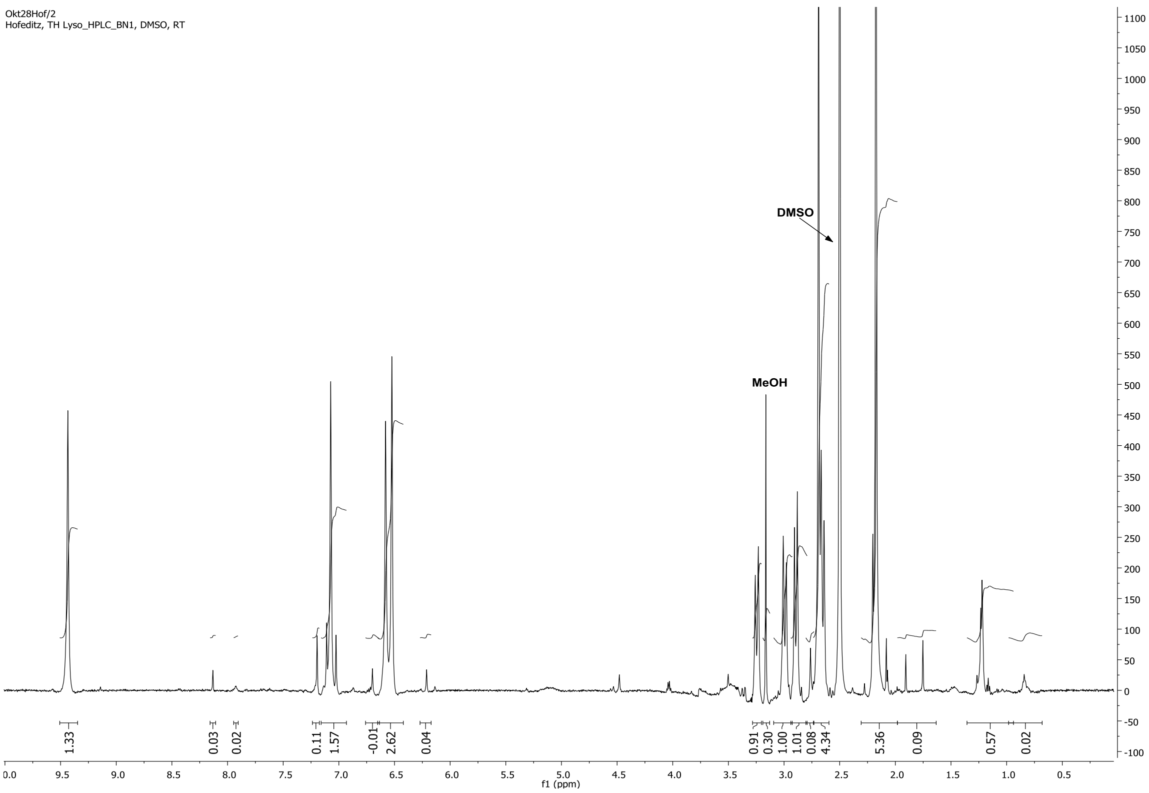

B

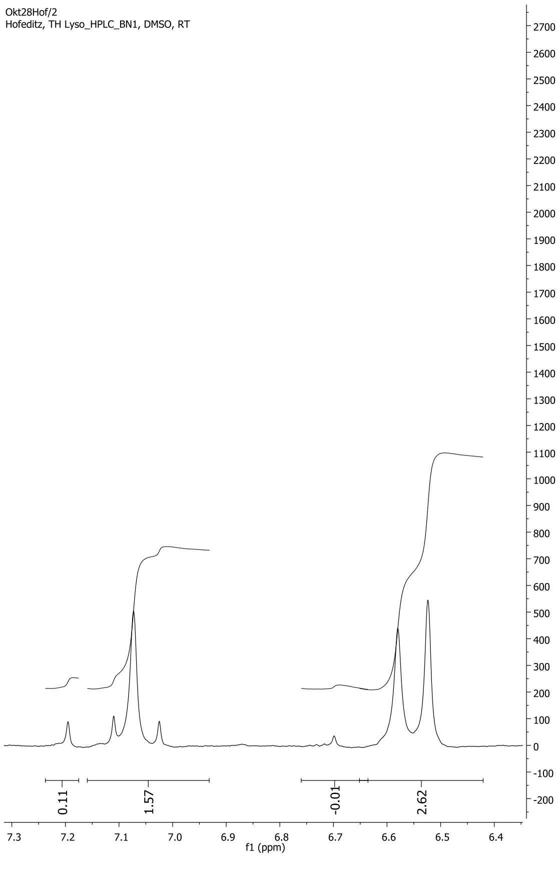

C

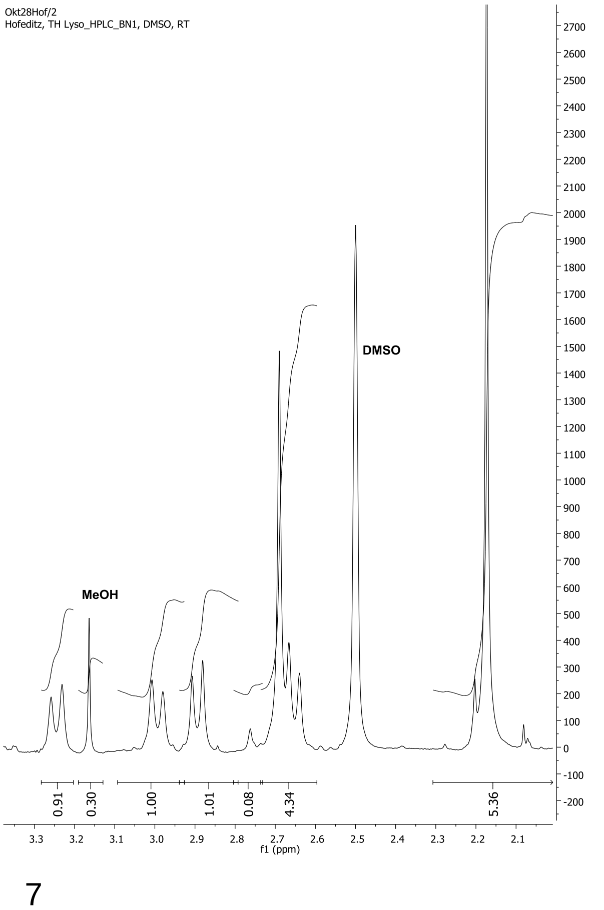

D

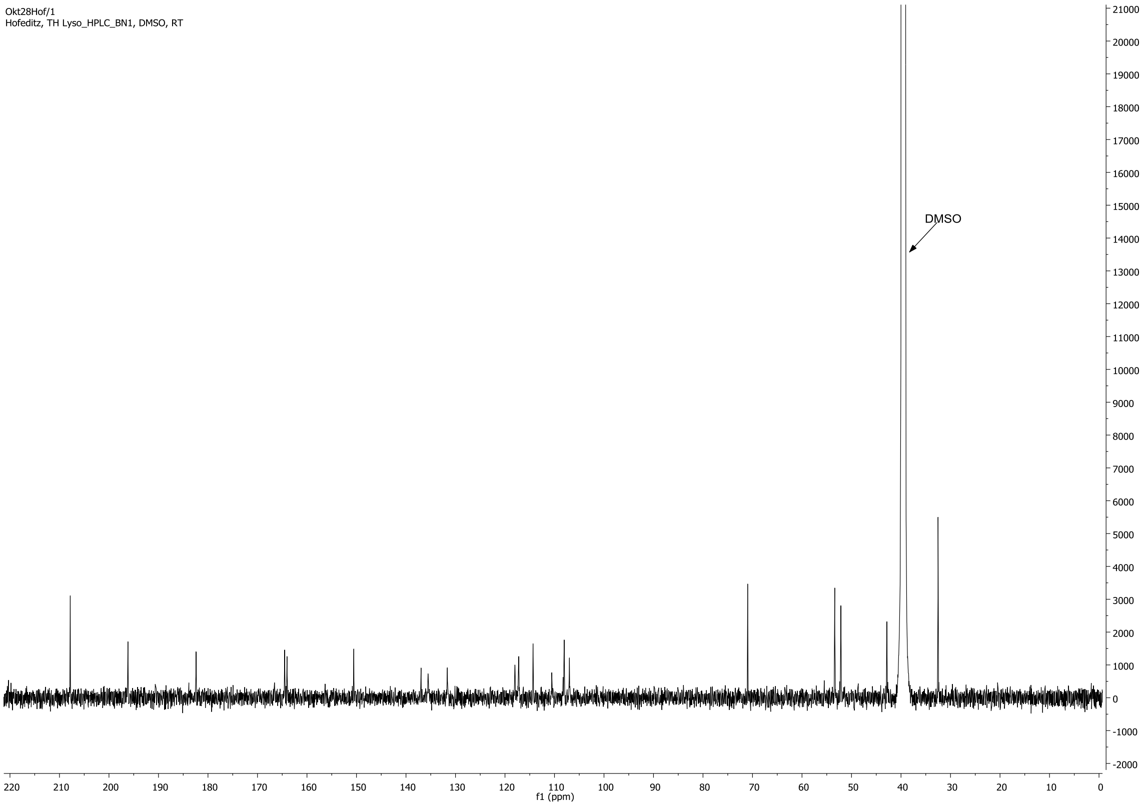

E

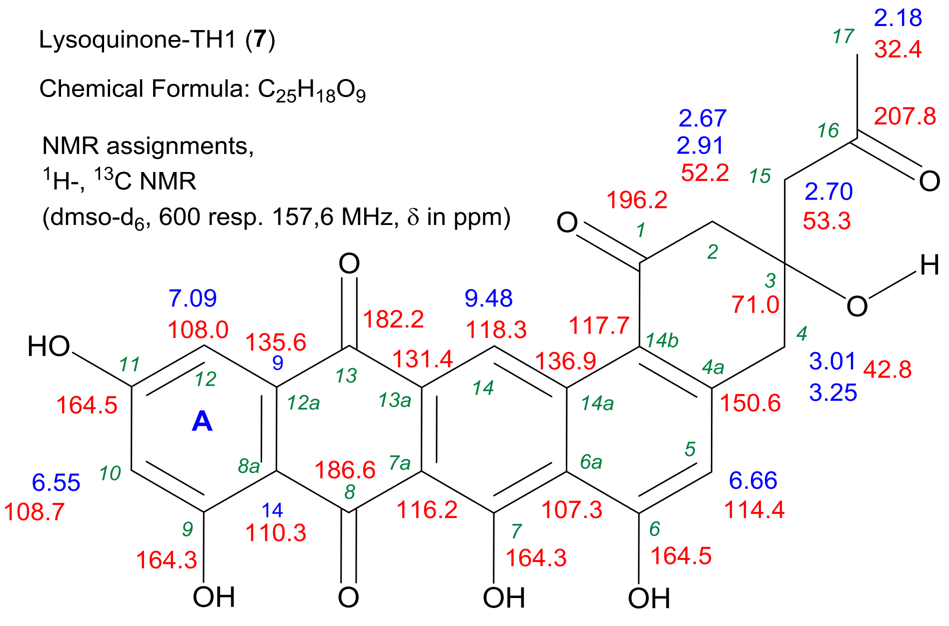

Figure S3: NMR spectra (600 resp. 157.6 MHz, DMSO-*d6*) of lysoquinone-TH1 (**7**).

A: ^{1}H-NMR spectrum of **7** (DMSO-*d6*, 600 MHz). **B:** Expansions of the ^{1}H-NMR spectrum of **7** in a range of δ_H = 6.4–7.3 ppm. **C:** Expansions of the ^{1}H-NMR of **7** in a range of δ_H = 2.1-3.3 ppm. **D:** ^{13}C-NMR of **7**. **E:** Detailed ^{1}H- and ^{13}C NMR assignments (δ in ppm).

6 Figure S4: NMR spectra of ^{13}C-enriched lysoquinone-TH1 (7)

(A)

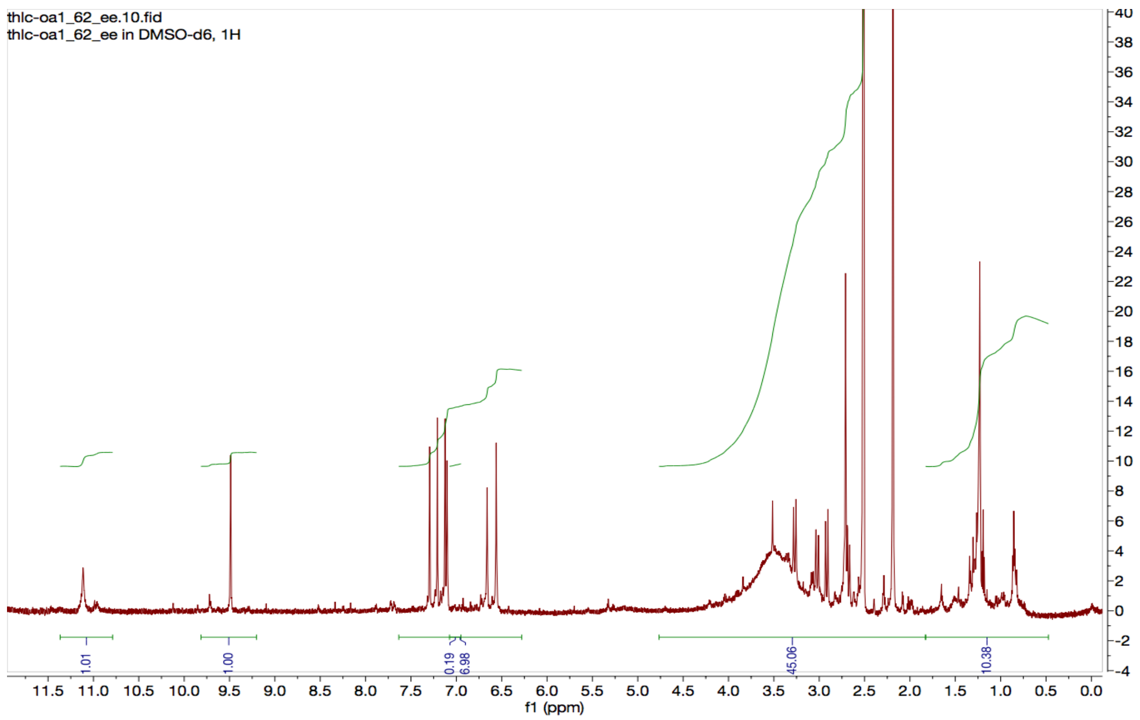

(B)

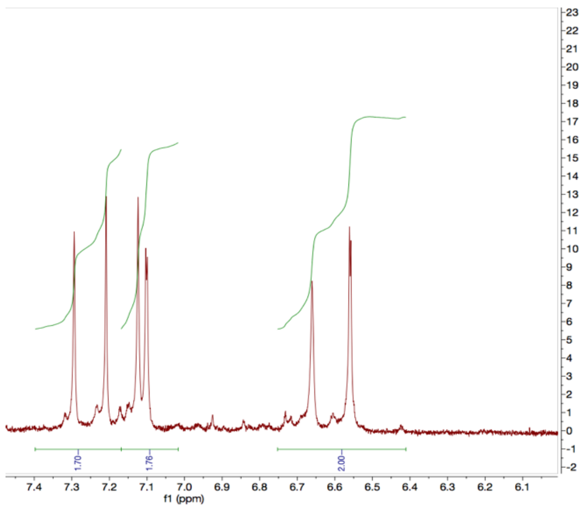

(C)

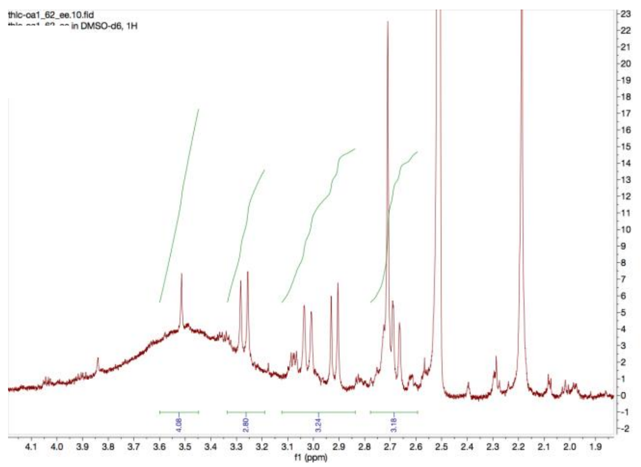

(D)

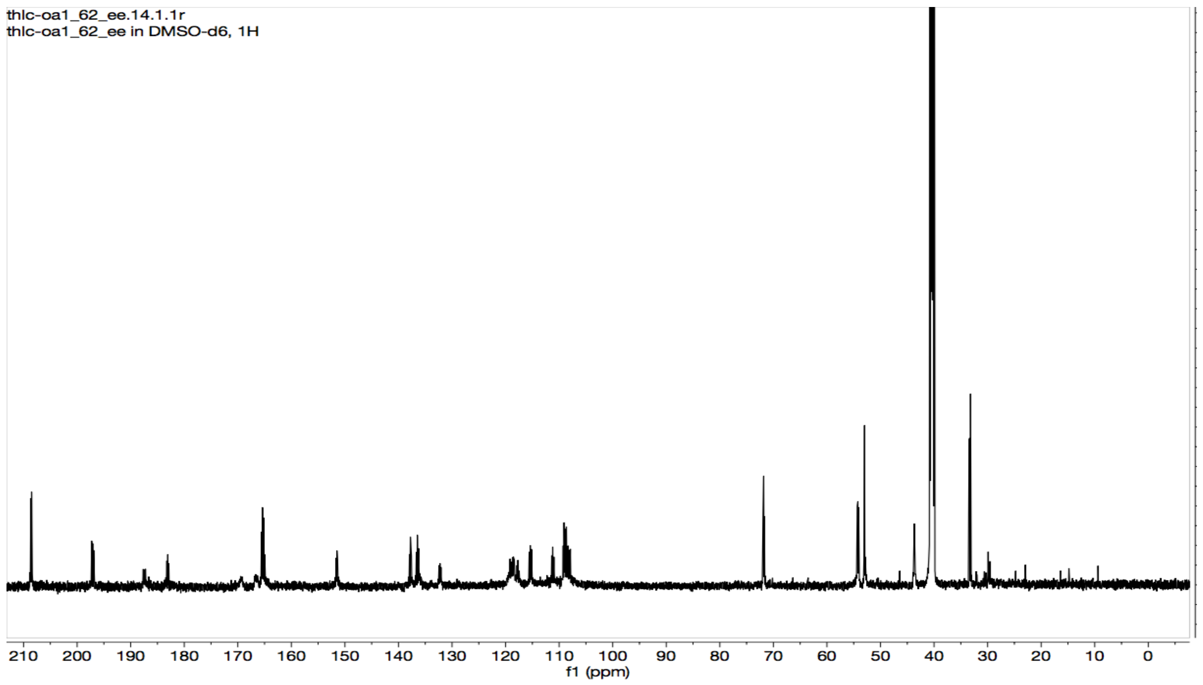
thlc-oa1_62_ee.14.1.1r
thlc-oa1_62_ee in DMSO-d6, 1H
210 200 190 180 170 160 150 140 130 120 110 100 90 80 70 60 50 40 30 20 10 0
f1 (ppm)

(E) to (O)

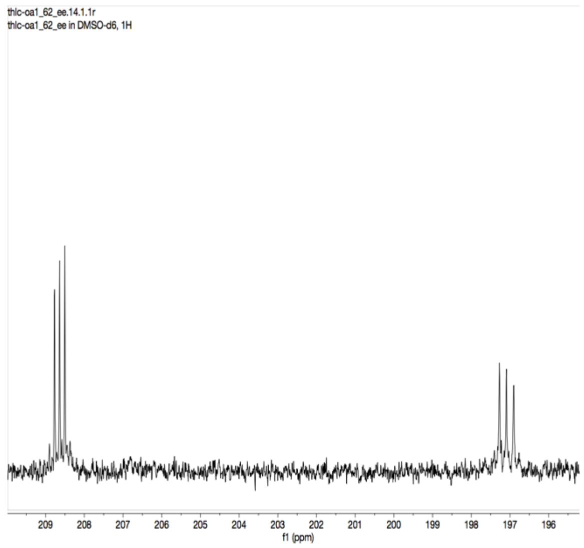
thlc-oa1_62_ee.14.1.1r
thlc-oa1_62_ee in DMSO-d6, 1H
209 208 207 206 205 204 203 202 201 200 199 198 197 196
f1 (ppm)

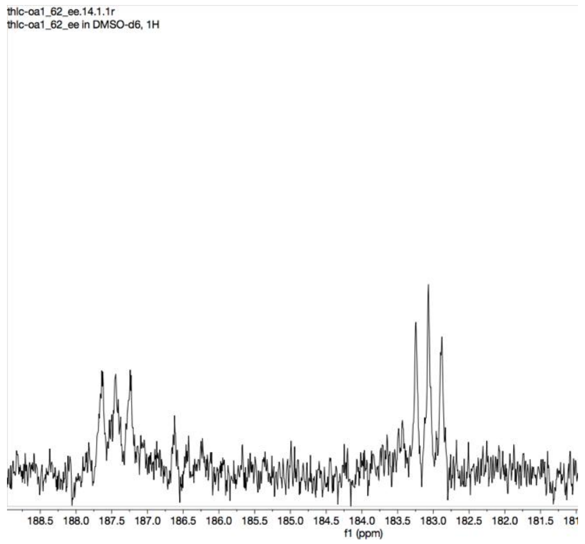
thlc-oa1_62_ee.14.1.1r
thlc-oa1_62_ee in DMSO-d6, 1H
188.5 188.0 187.5 187.0 186.5 186.0 185.5 185.0 184.5 184.0 183.5 183.0 182.5 182.0 181.5 181
f1 (ppm)

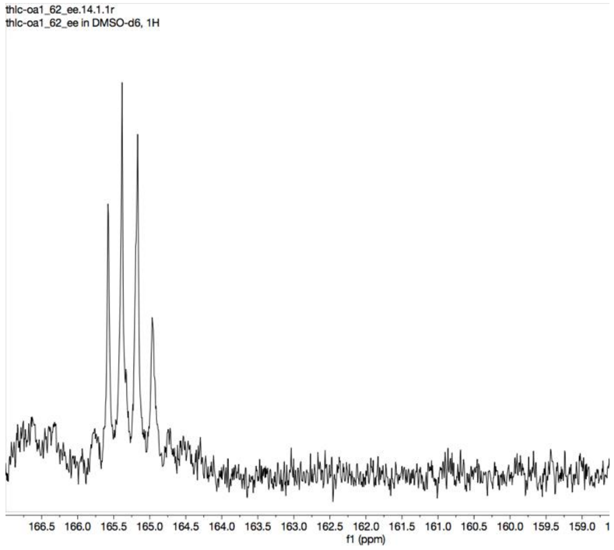
thlc-oa1_62_ee.14.1.1r
thlc-oa1_62_ee in DMSO-d6, 1H
166.5 166.0 165.5 165.0 164.5 164.0 163.5 163.0 162.5 162.0 161.5 161.0 160.5 160.0 159.5 159.0
f1 (ppm)

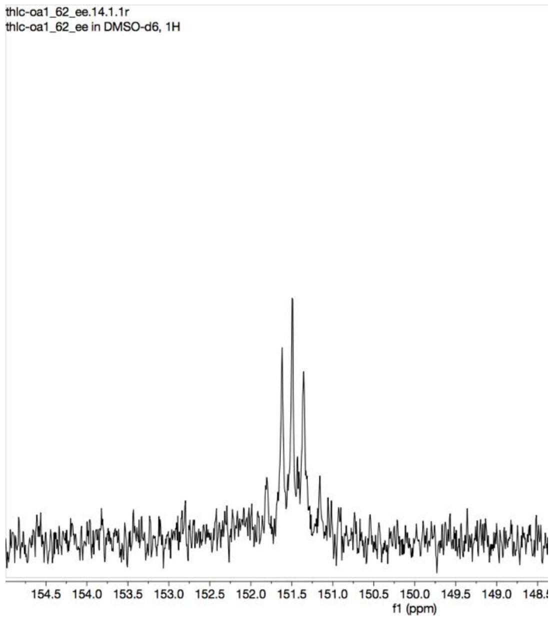
thlc-oa1_62_ee.14.1.1r
thlc-oa1_62_ee in DMSO-d6, 1H
154.5 154.0 153.5 153.0 152.5 152.0 151.5 151.0 150.5 150.0 149.5 149.0
f1 (ppm)

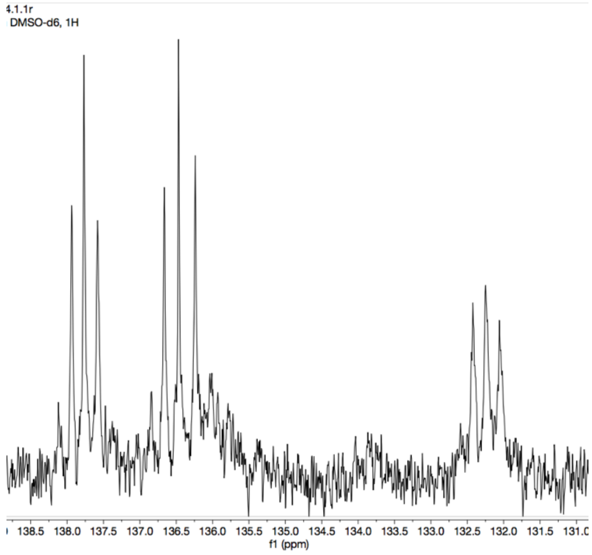
DMSO-d6, 1H
138.5 138.0 137.5 137.0 136.5 136.0 135.5 135.0 134.5 134.0 133.5 133.0 132.5 132.0 131.5
f1 (ppm)

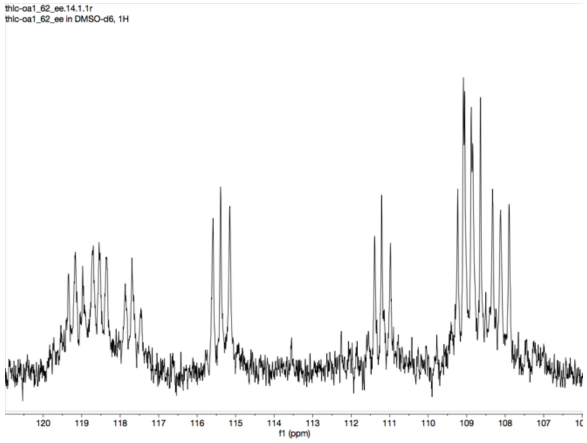
thlc-oa1_62_ee.14.1.1r
thlc-oa1_62_ee in DMSO-d6, 1H
120 119 118 117 116 115 114 113 112 111 110 109 108 107
f1 (ppm)

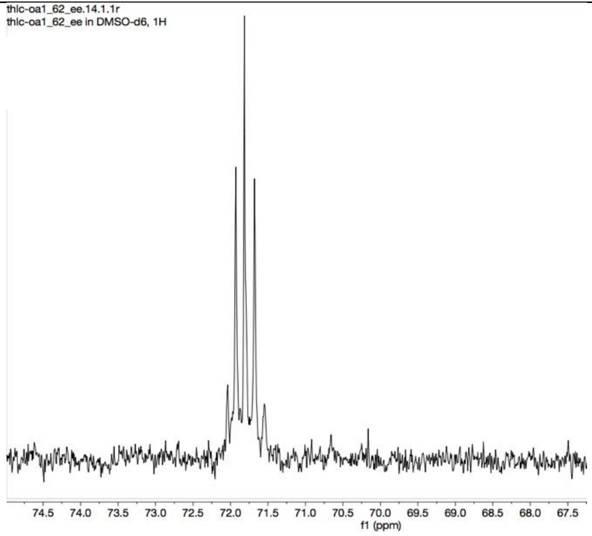
thlc-oa1_62_ee.14.1.1r
thlc-oa1_62_ee in DMSO-d6, 1H
74.5 74.0 73.5 73.0 72.5 72.0 71.5 71.0 70.5 70.0 69.5 69.0 68.5 68.0 67.5
f1 (ppm)

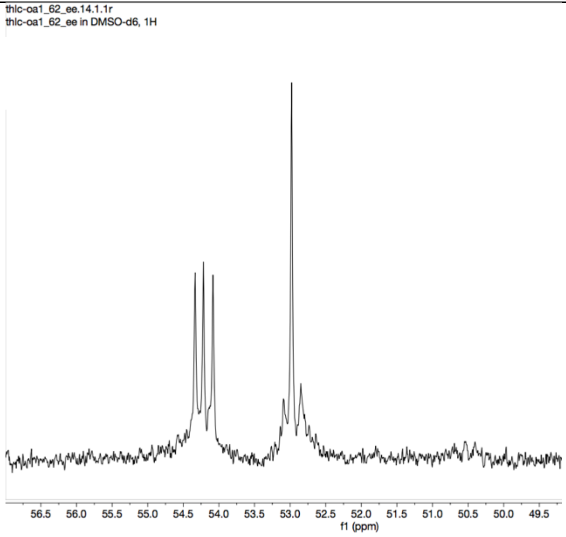
thlc-oa1_62_ee.14.1.1r
thlc-oa1_62_ee in DMSO-d6, 1H
56.5 56.0 55.5 55.0 54.5 54.0 53.5 53.0 52.5 52.0 51.5 51.0 50.5 50.0 49.5
f1 (ppm)

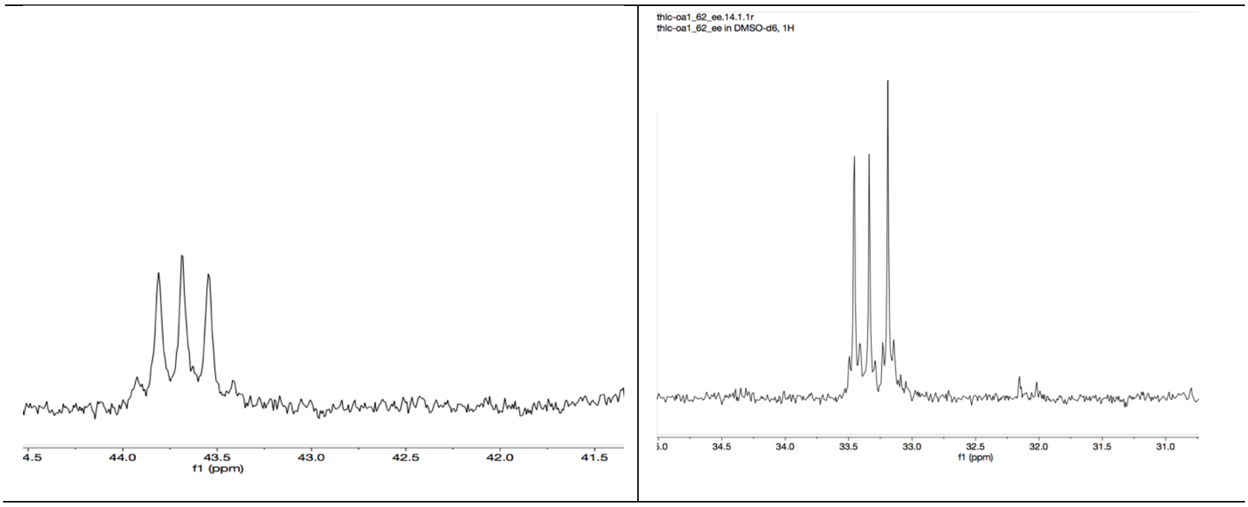

Figure S4: NMR spectra (600 resp. 157.6 MHz, DMSO-*d6*) of ^{13}C-enriched lysoquinone-TH1 (**7**) from doubly labeled [1,2-$^{13}C_2$]acetate feeding experiments.

A: ^{1}H-NMR (DMSO-*d6*; 600 MHz) of lysoquinone-TH1 (**7**) with ^{13}C-enrichment. **B:** Expansions of the ^{1}H-NMR of enriched **7** (δ_H = 6.1–7.5 ppm). **C:** Expansions of the ^{1}H-NMR of enriched **7** (δ_H = 2.60–3.60 ppm). **D:** ^{13}C-NMR (DMSO-*d6*; 150 MHz) of lysoquinone-TH1 (**7**) with ^{13}C-enrichment. **E:** Expansions of enriched **7** (δ_C = 195–210 ppm). **F:** Expansions of **7** (δ_C = 179–189 ppm). **G:** Expansions of **7** (δ_C = 157-167 ppm). **H:** Expansions of **7** (δ_C = 145–155 ppm). **I:** Expansions of **7** (δ_C = 130–140 ppm). **K:** Expansions of **7** (δ_C = 106–121 ppm). **L:** Expansions of **7** (δ_C = 65-75 ppm). **M:** Expansions of **7** (δ_C = 47–57 ppm). **N:** Expansions of **7** (δ_C = 41–46 ppm). **O:** Expansions of **7**(δ_C = 30–35 ppm).

7 Figure S5: 2D-NMR data of lysoquinone-TH1 (7)

Structure of lysoquinone-TH1 (7)

with important HMBC (heteronuclear multiple bond correlation) correlations from 2D-NMR-experiments

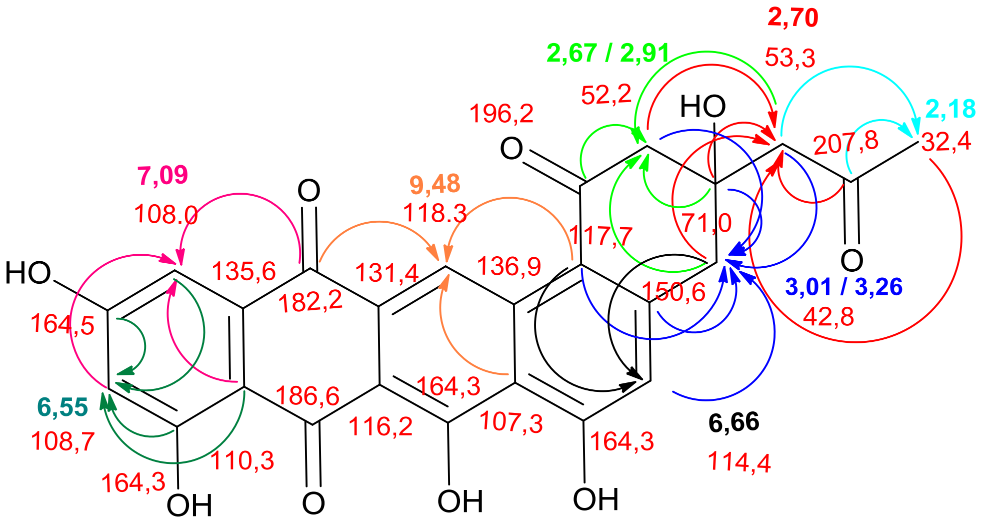

Figure S5: HMBC cross correlations of lysoquinone-TH1 (7) within the chemical shift of carbon atoms (red) and hydrogen atoms (different colors for enhanced overview). Depicted two-dimensional NMR-spectra with selected cross correlations: COSY-, HSQC-HMBC-experiment (next pages).

Figure S5A: COSY / HSQC correlations

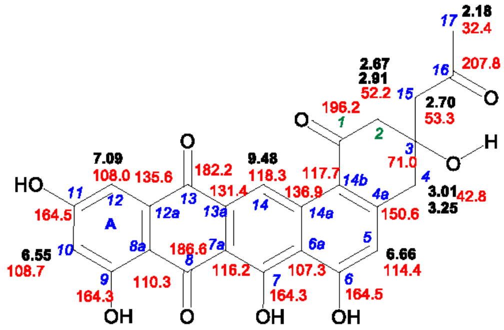

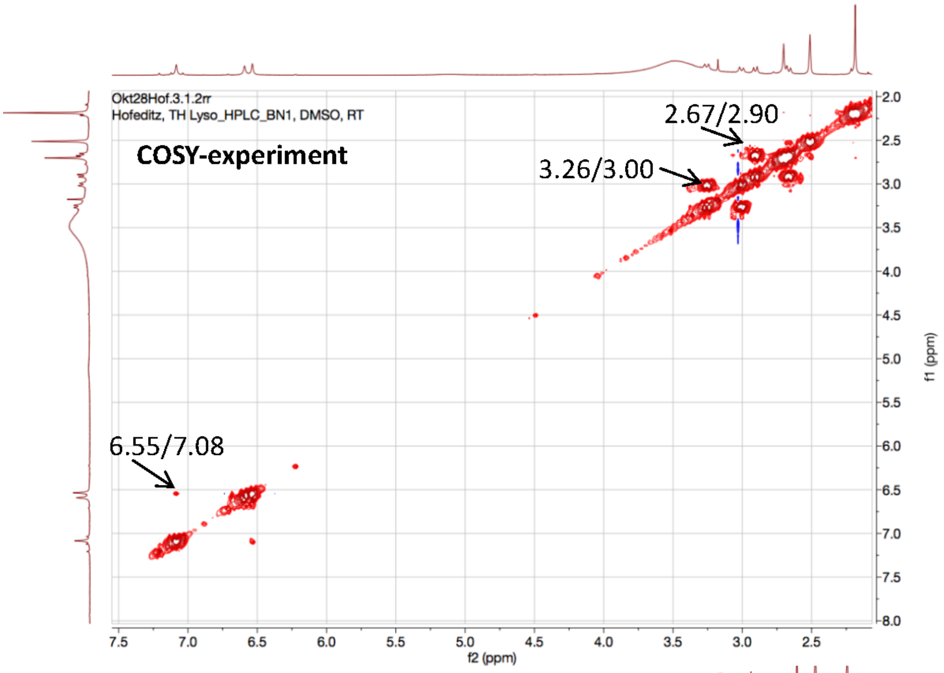

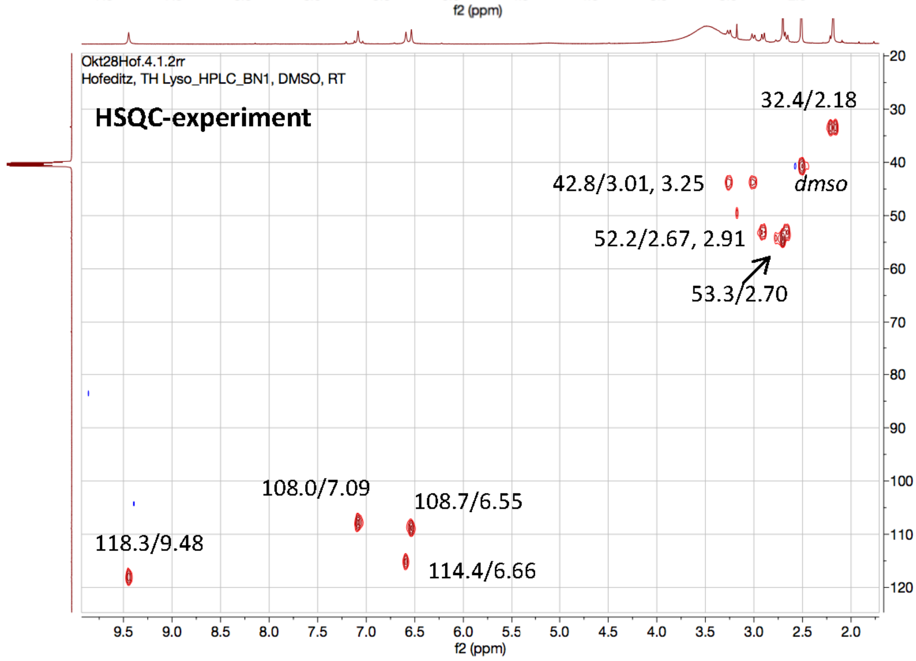

Figure S5B: HMBC correlations

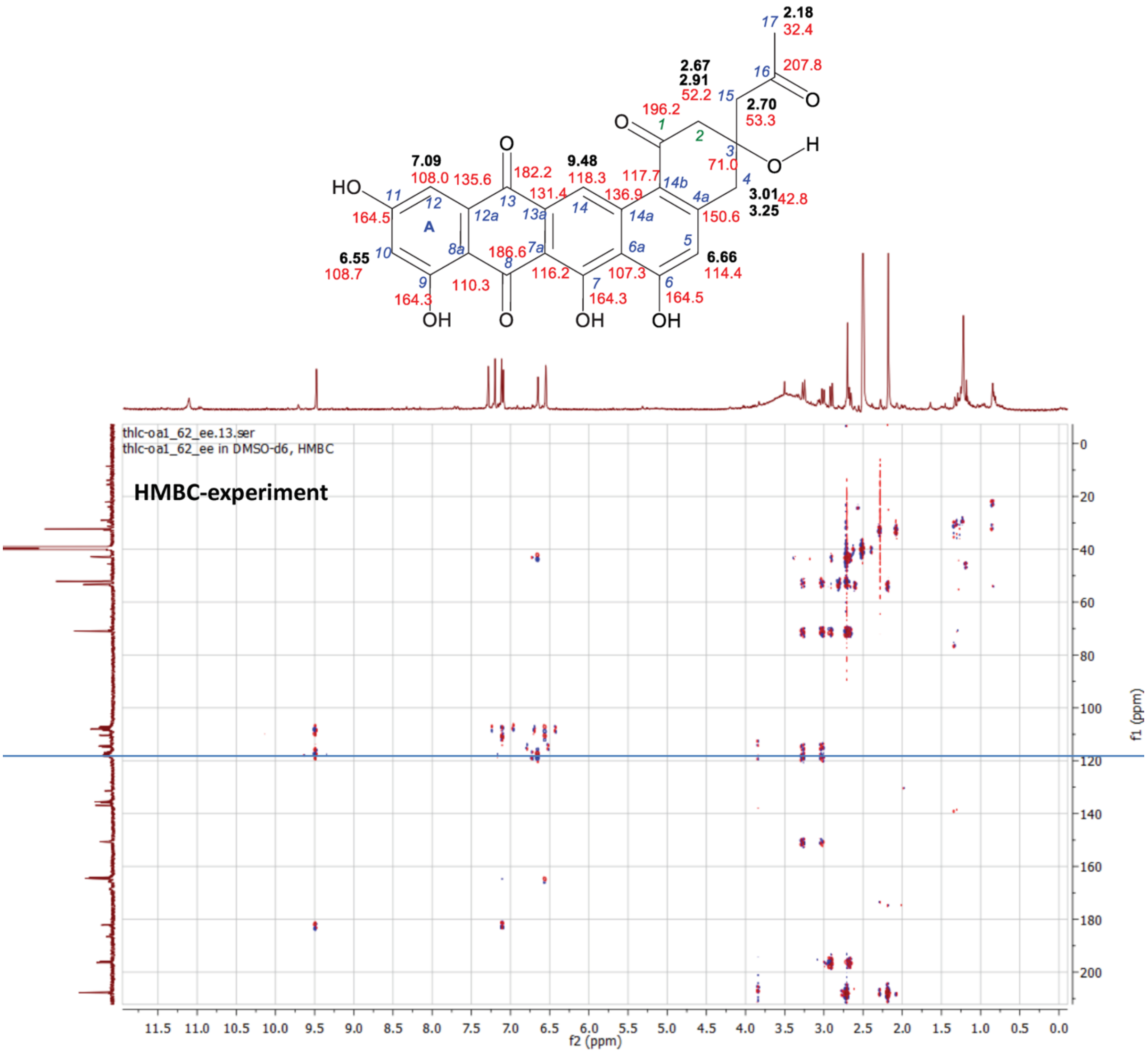

8 Figure S6: ^{13}C enrichment of 7: Structure, Table S1

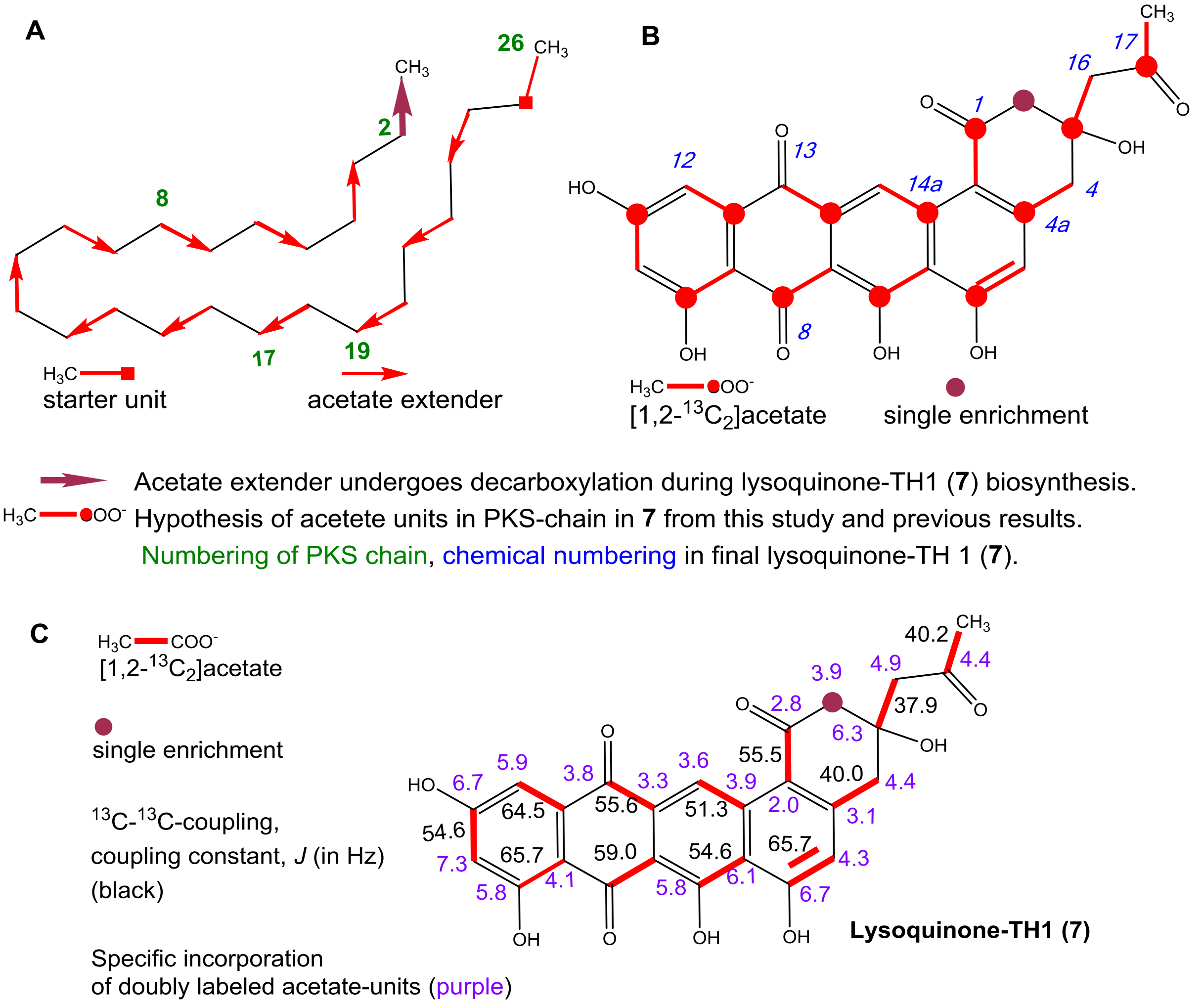

Figure S6: A: Suggested PKS chain of the tridecaketide as precursor of lysoquinone-TH1 (**7**).
B: Structural formula of **7** depicting intact acetate units from [1,2-$^{13}C_2$]-labeled acetate on the basis of this study and previous results (see references) **C:** Lysoquinone-TH1 (**7**) with assigned ^{13}C-^{13}C-coupling constants and specific incorporation (calculation, see below).

[1,2-$^{13}C_2$]-labeled acetate was purchased with 99% enrichment (*Cambridge Isotope laboratories, Inc.).* ^{13}C enrichment in the isolated lysoquinone-TH1 (**7**) was calculated as described by Scott *et al.*, *J. Am. Chem. Soc.* **1974**, *96*, 8069-8080:

Integrals of coupling signals of carbon atoms were calculated as the sum of the integral of the full multiplets. Assigned to the natural enrichment, the central signal of the methyl ^{13}C carbon atom C-17 of lysoquinone-TH1 (**7**) was used as the non-^{13}C-enriched internal reference signal.

Integrals for singlet signals of respective carbon atoms were calculated using the integral of the central line (I*CL*) of the multiplets and the usual singlet signals. All carbon atoms turned out to be enriched (Table S1, and see Fig S6 **C**).

$$\text{enrichment [\%]} = 1.1\% \times \frac{\text{integrated signal intensity (labeled compound)}}{\text{integrated signal intensity (reference)}} - 1.1\%$$

$$\text{specific incorporation} = \frac{\text{enrichment [\%]} \times 100}{\text{enrichment of precursor isotope [\%]}}$$

Table S1: Level of enrichment and specific incorporation of lysoquinone-TH1 (**7**), resulted from the feeding experiment with doubly labeled [1,2-$^{13}C_2$] acetate.

**C-atom**	δ_C [ppm]	**Integrated Intensity** (labeled compound)	**Integrated Intensity** (Reference)	**enrichment** [%]	**specific incor-poration**	**statistical coupling to**
**C-1**	196.2	125680.67	39478.23	0.03	2.8	C-2
**C-2**	52.2	272409.62	67517.08	0.04	3.9	C-1, C-3
**C-3**	71.0	239294.07	39925.74	0.06	6.3	C-2, C-4
**C-4**	42.8	252561.56	56466.40	0.04	4.4	C-3
**C-4a**	150.6	119859.13	35293.11	0.03	3.1	C-5
**C-5**	114.4	164560.28	38021.24	0.04	4.3	C-4a
**C-6**	164.5	230040.07	36491.45	0.07	6.7	C-6a
**C-6a**	107.3	155109.45	26769.51	0.06	6.1	C-62
**C-7**	164.3	148500.54	26779.16	0.06	5.8	C-7a
**C-7a**	116.2	92927.53	---	---	---	C-7
**C-8**	186.6	106031.71	---	---	---	C-8a
**C-8a**	110.3	121111.92	28617.71	0.04	4.1	C-8
**C-9**	164.3	148500.54	26779.16	0.06	5.8	C-10
**C-10**	108.7	258956.65	38026.93	0.07	7.3	C-9
**C-11**	164.5	230040.07	36491.45	0.07	6.7	C-12
**C-12**	108.0	218910.11	38897.04	0.06	5.9	C-11
**C-12a**	135.6	128030.79	32670.37	0.04	3.8	C-13
**C-13**	182.2	113731.76	28538.17	0.04	3.8	C-12a
**C-13a**	131.4	116592.22	32613.67	0.03	3.3	C-14
**C-14**	118.3	126176.64	33066.76	0.04	3.6	C-13a
**C-14a**	136.9	121559.05	29922.87	0.04	3.9	C-14b
**C-14b**	117.7	156522.22	62356.91	0.02	2.0	C-14a
**C-15**	53.3	281943.39	58169.04	0.05	4.9	C-16
**C-16**	207.8	162065.11	36747.45	0.04	4.4	C-15
**C-17**	32.4	328644.60	72761.44	0.00	0.0	---

9 Figure S7 – PDE-4B2 inhibition assay with lysoquinone-TH1

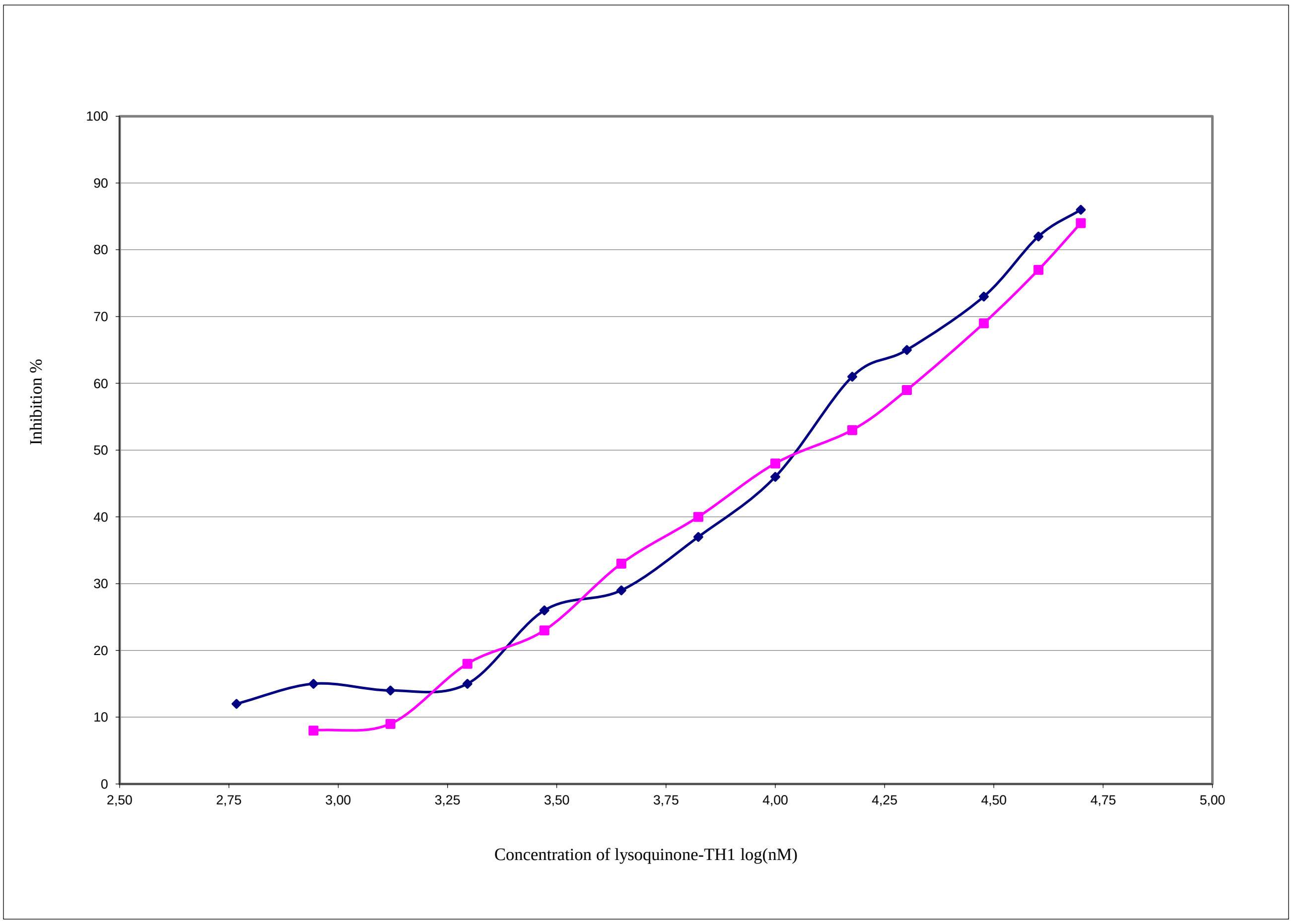

Figure S7: Inhibition assay of the enzyme PDE-4B2 by lysoquinone-TH1 (**7**). The results of two experiments are shown.
